# Intestinal Epithelial Cells and the Microbiome Undergo Swift Reprogramming at the Inception of Colonic Citrobacter rodentium Infection

**DOI:** 10.1128/mBio.00062-19

**Published:** 2019-04-02

**Authors:** Eve G. D. Hopkins, Theodoros I. Roumeliotis, Caroline Mullineaux-Sanders, Jyoti S. Choudhary, Gad Frankel

**Affiliations:** a Centre for Molecular Microbiology and Infection, Department of Life Sciences, Imperial College, London, United Kingdom; b Functional Proteomics Group, Chester Beatty Laboratories, Institute of Cancer Research, London, United Kingdom; GSK Vaccines

**Keywords:** cholesterol homeostasis, *Citrobacter rodentium*, host response to infection, intestinal epithelial cells, the microbiome

## Abstract

We used the mouse attaching and effacing (A/E) pathogen Citrobacter rodentium, which models the human A/E pathogens enteropathogenic Escherichia coli and enterohemorrhagic E. coli (EPEC and EHEC), to temporally resolve intestinal epithelial cell (IEC) responses and changes to the microbiome during *in vivo* infection. We found the host to be unresponsive during the first 3 days postinfection (DPI), when C. rodentium resides in the caecum. In contrast, at 4 DPI, the day of colonic colonization, despite only sporadic adhesion to the apex of the crypt, we observed robust upregulation of cell cycle and DNA repair processes, which were associated with expansion of the crypt Ki67-positive replicative zone, and downregulation of multiple metabolic processes (including the tricarboxylic acid [TCA] cycle and oxidative phosphorylation). Moreover, we observed dramatic depletion of goblet and deep crypt secretory cells and an atypical regulation of cholesterol homeostasis in IECs during early infection, with simultaneous upregulation of cholesterol biogenesis (e.g., 3-hydroxy-3-methylglutaryl–coenzyme A reductase [Hmgcr]), import (e.g., low-density lipoprotein receptor [Ldlr]), and efflux (e.g., AbcA1). We also detected interleukin 22 (IL-22) responses in IECs (e.g., Reg3γ) on the day of colonic colonization, which occurred concomitantly with a bloom of commensal Enterobacteriaceae on the mucosal surface. These results unravel a new paradigm in host-pathogen-microbiome interactions, showing for the first time that sensing a small number of pathogenic bacteria triggers swift intrinsic changes to the IEC composition and function, in tandem with significant changes to the mucosa-associated microbiome, which parallel innate immune responses.

## Abstract (precis)

The mouse pathogen C. rodentium is a widely used model for colonic infection and has been a major tool in fundamental discoveries in the fields of bacterial pathogenesis and mucosal immunology. Despite extensive studies probing acute C. rodentium infection, our understanding of the early stages preceding the infection climax remains relatively undetailed. To this end, we apply a multiomics approach to resolve temporal changes to the host and microbiome during early infection. Unexpectedly, we found immediate and dramatic responses occurring on the day of colonic infection, both in the host intestinal epithelial cells and in the microbiome. Our study suggests changes in cholesterol and carbon metabolism in epithelial cells are instantly induced upon pathogen detection in the colon, corresponding with a shift to primarily facultative anaerobes constituting the microbiome. This study contributes to our knowledge of disease pathogenesis and mechanisms of barrier regulation, which is required for development of novel therapeutics targeting the intestinal epithelium.

## INTRODUCTION

The intestinal epithelium serves a dual role as it enables nutrient absorption while simultaneously providing a barrier to commensal bacteria and pathogens (1). Constant renewal of the epithelium every 5 to 7 days is enabled by LGR5^+^ stem cells at the base of the crypts, where they lie intermingled with deep crypt secretory (DCS) cells called Reg4^+^ cells (2). Transit-amplifying (TA) cells, arising from proliferation and partial differentiation of LGR5^+^ cells, rapidly divide in the lower half of the crypt a number of times before arresting their cell cycle and differentiating into mature cell types as they migrate to the crypt’s upper surface. These differentiated cell types include absorptive enterocytes, goblet cells, enteroendocrine cells, and tuft cells. In addition to providing a barrier, intestinal epithelial cells (IECs) also have immunoregulatory properties enabling them to detect invading pathogens, e.g., they are able to express pattern recognition receptors, and subsequently influence development of the mucosal immune cell response (3).

Citrobacter rodentium, an extracellular mouse pathogen, is a physiologically relevant model for the human clinical pathogens enteropathogenic Escherichia coli (EPEC) and enterohemorrhagic E. coli (EHEC), and it has been widely used to probe mucosal responses to colonic infection (4, 5). Hallmarks of C. rodentium infection include tissue regeneration via colonic crypt hyperplasia (CCH), which results from increased amplification of TA cells in conjunction with inhibition of both anoikis and cell detachment. (5). Furthermore, the host has been shown to mount a robust nutritional immune response to C. rodentium infection, manifested by secretion of lipocalin-2 (LCN-2) and calprotectin (a heterodimer of subunits S100A8 and S100A9), which sequester the trace minerals Fe (LCN-2) and Mn and Zn (calprotectin) (6).

Following oral inoculation, C. rodentium initially colonizes the cecal patch, a major lymphoid structure in the cecum, where it adapts to the gut environment (7). We define these first few days as the establishment phase, during which most of the inoculum passes straight through the intestinal tract and is shed in the feces (8). C. rodentium spreads from the cecal patch to the colon at 4 days postinfection (DPI), enabling rapid bacterial proliferation as it penetrates the mucosa and intimately attaches to IECs, which we define as the expansion phase. Colonization levels plateau at 10^8^ to 10^9^ CFU/g of feces between 8 to 12 DPI, before bacterial shedding decreases as the infection starts to clear between 12 to 16 DPI, which we define as the steady-state and clearance phases, respectively (5, 8). Mice develop colitis during C. rodentium infection (4), and dysbiosis is induced in the large intestine, resulting in a reduction in the overall abundance and diversity of commensal bacteria (9).

IECs express the receptor for interleukin 22 (IL-22), which plays an essential role during C. rodentium infection, as it fortifies the intestinal barrier and restricts the pathogen to the gut (10). IL-22 also promotes production of antimicrobial peptides (AMPs), e.g., Reg3β and Reg3γ, LCN-2, calprotectin, and mucins (11–14). IL-22 is produced by innate class 3 lymphoid cells (ILC3s) during the establishment and expansion phases of infection, followed by CD11b^+^ Ly6C^+^ Ly6G^+^ neutrophils (15) and Th17 and Th22 T cells (16, 17), which contribute to IL-22 production during the steady-state and clearance phases. Despite IL-22 promoting epithelial regeneration in a STAT-3-dependent manner, *IL-22^–/–^* mice exhibit increased CCH and tissue damage at peak C. rodentium infection compared to that of wild-type mice (18), suggesting that in the absence of IL-22 there is an uncoordinated damage repair response to infection. Furthermore, IL-22 has also been implicated in regulating tight junctions and the permeability of IECs, including upregulation of the paracellular water and Na^+^ channel Claudin-2 (19, 20).

Recently, we reported that C. rodentium subverts IEC metabolism in order to evade innate immune responses and establish a favorable niche in the colon (6, 21). Using multiplex proteomics, we uncovered a significant downregulation of host metabolic pathways, including gluconeogenesis, lipid metabolism, the tricarboxylic acid (TCA) cycle and oxidative phosphorylation (OXPHOS), with a simultaneous upregulation of cell cycle and DNA replication pathways. At this stage, the IECs seemed to rely on aerobic glycolysis, fueled by a robust upregulation of the basolateral glucose transporter Slc5A9 (6). Presumably, this metabolic reprogramming occurs to meet the increased cellular energetic demands mediating tissue repair responses to the infection. Furthermore, our proteomics data, validated with a fecal cholesterol quantification assay, highlighted upregulation of both cholesterol biogenesis and efflux/influx pathways (6), processes which are usually regulated antagonistically. These observations are consistent with cholesterol being an essential ingredient of new membranes formed in proliferating cells and having a role in innate immunity.

Importantly, while studies of pathogen-host interactions are usually conducted at the acute phase of the infection, little is currently known about the temporal host responses culminating before the pathogen burden peaks. The aim of this study was to temporally resolve IEC and microbiome responses at the expansion phase of C. rodentium infection (4 and 6 DPI). This revealed a dramatic reprogramming of the crypt cellular composition, metabolism, and DNA replication and repair immediately as the pathogen starts to colonize the colon, which coincided with the expansion of mucosa-associated Enterobacteriaceae.

## RESULTS

### Sporadic mucosal association of C. rodentium at the onset of colonic colonization.

We recently reported IEC responses to C. rodentium at the steady-state phase of the infection (8 DPI), including reprogramming of bioenergetics and metabolism (6). Here, we aimed to track the progression of C. rodentium infection-induced alterations to the gut microenvironment, using multiplexed quantitative proteomics, transcriptomics, enzyme-linked immunosorbent assay (ELISA), and 16S rRNA gene sequencing.

**FIG 1:**
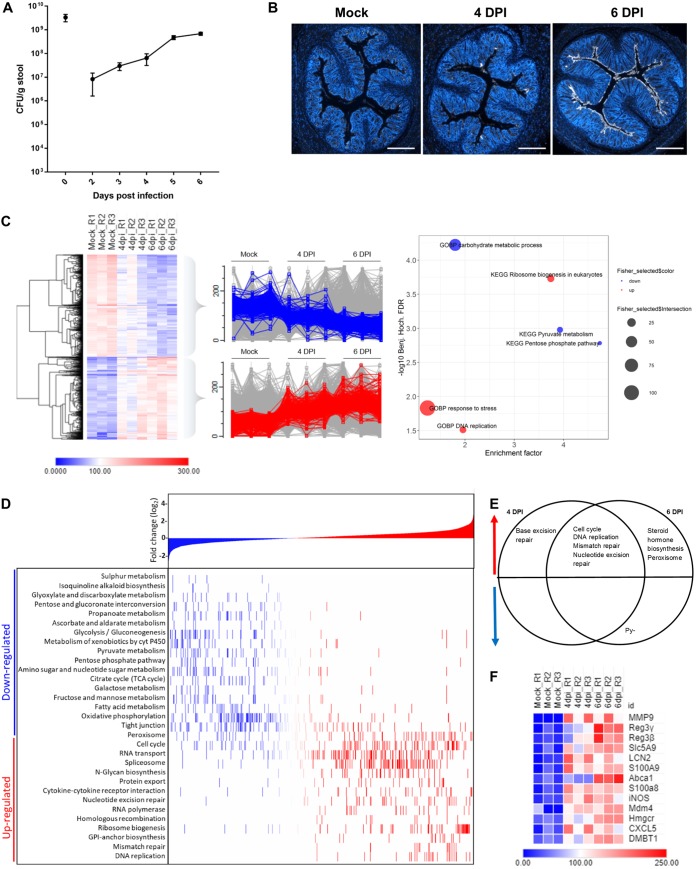
Sporadic C. rodentium colonization at 4 DPI induces upregulation of the cell cycle and downregulation of metabolic processes. (A) Line graph showing average numbers of CFU/g feces with standard error of the mean bars over the time course of C. rodentium infection (*n* = 10). (B) Representative images of immunostaining of C. rodentium (white) and DNA (blue) on colonic sections (*n* = 10) from mock-infected (Mock) or infected mice at 4 DPI or 6 DPI, as indicated. Sections from 4 DPI were highly varied; thus, the image with the average level of C. rodentium staining was selected. Scale bar = 500 µm. (C) Heat map showing proteins with significantly altered abundances at 6 DPI compared to abundances in mock-infected mice and filtered for the 30% most altered proteins upon infection. Scaled abundances for 3 biological repeats of each time point are shown (R1 to R3). Profile plots show significant downregulated proteins in blue and upregulated proteins in red. The right-most plot shows relative enrichments of named KEGG and GOBP pathways. Fischer exact test FDR, <0.05. Benj. Hoch., Benjamini-Hochberg. (D) KEGG pathway enrichment analysis. Proteins are ranked according to log_2_ values on the *x* axis, with increasingly negative log_2_FC values on the far left (blue) to increasingly positive log_2_FC values during infection on the far right (red). 1D annotation enrichment of KEGG pathways during infection are highlighted in the heat map on the *y* axis, with the most downregulated pathways at the top to the most upregulated pathways at the bottom. FDR < 0.05; *t* test (*n* = 3). (E) Venn diagram showing pathways that are upregulated (red) or downregulated (blue) at 4 DPI, 6 DPI, or both time points of infection. (F) Heat map of selected proteins from the top 100 proteins when statistically significantly altered abundances are ranked based on the highest fold change at 6 DPI compared to abundances in mock-infected controls.

We first performed temporal profiling of C. rodentium shedding and tissue association during the expansion phase of infection. This revealed that shedding reached an average of 6.5 × 10^7^ CFU/g by 4 DPI and 6.9 × 10^8^ CFU/g by 6 DPI (Fig. 1A). Importantly, we recorded larger standard errors of the means at 2 to 4 DPI than at 5 and 6 DPI, suggesting that while migrating from the cecum to the colon, C. rodentium is more sensitive to variable host environments in individual mice (e.g., the composition of the microbiota) but is able to adapt once initial colon colonization has occurred, thus reaching homogenous levels of colonization from 5 DPI onwards. Comparing tissue distributions of C. rodentium at 4 DPI (i.e., on the day of colonic colonization) and 6 DPI (i.e., the intermediate time between the expansion and steady-state phases) by immunohistochemistry revealed distinct differences in bacterial abundance and distribution (Fig. 1B). Notably, no C. rodentium was detected in colonic sections at 3 DPI (see Fig. S1 in the supplemental material). While binding of C. rodentium to the colonic mucosa was scarce and highly varied between different mice at 4 DPI, a uniform distribution of the pathogen along the entire colonic mucosa was seen at 6 DPI (Fig. S1). These data suggest that despite 3 × 10^7^ CFU/g being shed in the feces at 3 DPI, C. rodentium is not yet visibly detected in the large intestine; however, on the 4th day, C. rodentium is seeded in the colon, which is followed by rapid expansion.

10.1128/mBio.00062-19.1 FIG S1 Representative images of immunostaining of C. rodentium (white) and DNA (blue) in colon sections from mice (*n* = 10) mock infected or infected with C. rodentium at 3, 4, or 6 DPI, as indicated. *, the same sample as appears in Fig. 1B to enable comparison between different mice. Scale bar = 500 µm. Download FIG S1, TIF file, 1.4 MB. Copyright © 2019 Hopkins et al. 2019 Hopkins et al. This content is distributed under the terms of the Creative Commons Attribution 4.0 International license.

### C. rodentium triggers rapid polarization of metabolic and cell proliferation processes.

In order to determine the temporal impact of C. rodentium infection on IECs during the expansion phase, we performed proteomic analysis of colonic IECs isolated from C. rodentium*-*infected mice at 4 and 6 DPI using mock-infected mice as controls. We included 5 mice per time point and monitored colonization levels up until and including the day of extraction; mice that did not reach the minimum number of CFU per gram of stool thresholds (1 × 10^7^ CFU/g stool by 4 DPI or 1 × 10^8^ CFU/g stool by 6 DPI) were excluded from further processing to reduce heterogeneity within each condition (Fig. S2). Protein extracts from IECs enriched from mice within the same condition of a single biological repeat were pooled at a 1:1 ratio and labeled with tandem mass tags (TMT) before undergoing liquid chromatography-tandem mass spectrometry (LC-MS/MS) analysis (Fig. S3A; Table S1). To generate a robust data set, three biological repeats were included within the same multiplex experiment.

10.1128/mBio.00062-19.2 FIG S2 CFU-per-gram measurements were taken for mice used for the proteomics experiment. Mice that did not reach colonization levels of 1 × 10^7^ by 4 DPI or 1 × 10^8^ by 6 DPI were excluded from further processing to reduce heterogeneity within conditions (shown by the dotted line). Download FIG S2, TIF file, 1.2 MB. Copyright © 2019 Hopkins et al. 2019 Hopkins et al. This content is distributed under the terms of the Creative Commons Attribution 4.0 International license.

10.1128/mBio.00062-19.3 FIG S3 Two groups of mice (*n* = 5) were inoculated with ∼1 × 10^9^ C. rodentium organisms, and one group of mice (*n* = 5) was simultaneously mock inoculated with PBS. (A) Intestinal epithelial cells were enriched from the mice at either 4 DPI or 6 DPI, and individual mice were pooled within the group at a 1:1 protein ratio. This was repeated twice more as independent biological repeats. Each separate condition/biological repeat was then labeled with a different TMT reagent and subjected to LC-MS/MS analysis. (B) Volcano plot of log_2_(FC) versus the –log_10_(*P* value) showing the differential regulation of proteins in IECs during infection (combined 4- and 6-DPI data) compared to that of mock-infected samples. Download FIG S3, TIF file, 1.3 MB. Copyright © 2019 Hopkins et al. 2019 Hopkins et al. This content is distributed under the terms of the Creative Commons Attribution 4.0 International license.

10.1128/mBio.00062-19.9 TABLE S1 TMT labels, pooled samples, and mice selected for proteomics analysis. Mice that did not reach the colonization threshold set were excluded from processing. Download Table S1, PDF file, 0.01 MB. Copyright © 2019 Hopkins et al. 2019 Hopkins et al. This content is distributed under the terms of the Creative Commons Attribution 4.0 International license.

We quantified a total of 10,418 proteins, of which 9,127 were mapped to Mus musculus and 1,290 to C. rodentium (peptide false-discovery rate [FDR], <1%). Statistical analysis considering both the 4 and the 6 DPI data as infected samples identified 587 upregulated and 446 downregulated proteins compared to protein expression in the mock-infected samples (Fig. S3B). Further analysis of the most significantly changed proteins upon infection revealed two protein subsets: those that decrease during the course of infection, which highlighted processes, including carbohydrate and pyruvate metabolism, as downregulated, and those that increase, which showed upregulation of processes, including response to stress, DNA replication, and ribosome biogenesis (Fig. 1C). Bioinformatic analysis of the data set as a whole identified additional enriched pathways, including significant downregulation of further metabolic processes, such as the TCA cycle, OXPHOS, propanoate, pyruvate, and starch and glucose metabolism, and upregulation of cell cycle and DNA repair pathways (mismatch repair, homologous recombination, and nucleotide excision repair) (Fig. 1D). Unexpectedly, many of these processes correlate with those identified as significantly altered at the steady-state phase of infection (6), suggesting that a significant response to pathogen infection is mounted in IECs during the expansion phase. Interestingly, Forkhead box O3 (FOXO3), a transcription factor involved in modulating the metabolic state and cellular apoptosis, was predicted to be significantly inactivated during C. rodentium infection (enrichment score, −0.31; Benjamini-Hochberg FDR, 8.16E–03), correlating with a previous *in vitro* study that showed that C. rodentium infection led to inactivation of FOXO3 in intestinal epithelia (Fig. S4) (22).

10.1128/mBio.00062-19.4 FIG S4 Bar plot showing targets of the named transcription factor in red, if it increased in abundance at 4 DPI, and in blue if it decreased in abundance at 4 DPI. Enrichment scores are shown in gray on the right axis. Targets are presented as percentages of the total number of targets of the transcription factor in question. Download FIG S4, TIF file, 0.8 MB. Copyright © 2019 Hopkins et al. 2019 Hopkins et al. This content is distributed under the terms of the Creative Commons Attribution 4.0 International license.

To further resolve temporal changes, one-dimensional (1D) enrichment analysis was applied to 4 and 6 DPI samples separately, revealing early onset of a number of DNA repair pathways, with base excision repair specific to 4 DPI only (Fig. 1E). Significant upregulation of a number of DNA repair pathways at 4 DPI, in addition to upregulation of cell cycle and DNA replication processes, strongly suggests that proliferation pathways are activated even when C. rodentium colonization is low, sporadic, and restricted to the upper surface of the crypt. While both expansion phase time points show downregulation of the TCA cycle, the pentose phosphate pathway, and pyruvate metabolism, only 6 DPI additionally shows upregulation of steroid hormone biosynthesis, suggesting that changes to IEC metabolism are initiated as early as the day of colonic colonization (4 DPI), but further aspects of metabolism become significantly affected as pathogen levels in the colon increase. Furthermore, downregulation of the TCA cycle and OXPHOS suggests a progressive shift of cellular bioenergetics during infection to aerobic glycolysis, which coincides with an increase in cell proliferation.

On a protein-specific level, among the proteins ranked in the top 100 for most changed in abundance, we found a number of innate immunity and nutritional immunity proteins, including matrix metallopeptidase 9 (MMP9), Reg3β, Reg3γ, inducible nitric oxide synthase (iNOS), LCN-2, DMBT1, S100A8, and S100A9 (Fig. 1F). Furthermore, the neutrophil chemoattractant CXCL5, the glucose transporter that fuels glycolysis, Slc5A9, the rate-limiting enzyme in the cholesterol biogenesis pathway, Hmgcr (3-hydroxy-3-methylglutaryl–coenzyme A reductase), and the basolateral cholesterol efflux transporter Abca1 were also found among top 100 proteins with significantly increased abundance during infection. Together, these data show that a significant change in both the metabolic and proliferative states of IECs is induced on the day of colonic colonization (4 DPI), coinciding with innate and nutritional immunity responses.

### Cholesterol biogenesis and efflux are simultaneously induced at the expansion phase.

**FIG 2:**
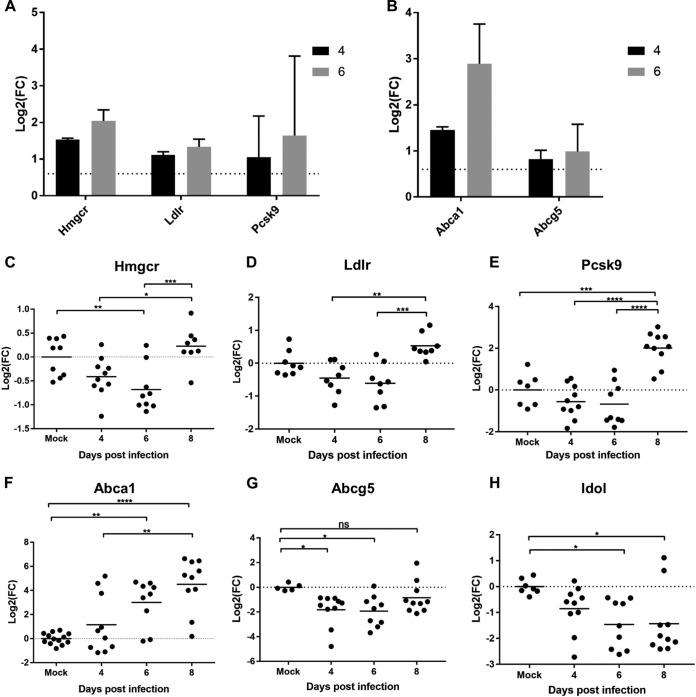
Cholesterol homeostasis is perturbed during early infection. (A and B) Log_2_FCs in infected samples (4 or 6 DPI, as indicated) compared with samples from mock-infected mice of named SREBP2-induced proteins (A) and LXR-induced proteins (B). The dotted line at log_2_FC value 0.6 represents the implicated cutoff for an upregulated protein. (C to H) A qRT-PCR analysis of *Hmgcr* (C), *Ldlr* (D), *Pcsk9* (E), *Abca1* (F), *Abcg5* (G), and *Idol* (H) revealed expression levels in IECs enriched from mock-infected or C. rodentium-infected colons. Shown are log_2_FCs in expression levels compared to the average level in mock-infected mice from the same biological repeat. Statistical significance was determined by Tukey’s multiple-comparison test. *, *P* < 0.05; **, *P* < 0.01; ***, *P* < 0.001; ****, *P* < 0.0001; ns, not significant.

In addition to Hmgcr, the other proteins in the cholesterol biogenesis cascade (Fig. S5A) and the low-density lipoprotein receptor (Ldlr), as well as proprotein convertase subtilisin/kexin type 9 (Pcsk9), which antagonizes Ldlr (Fig. 2A), were found in increased abundance during the expansion phase of infection compared to their levels in mock-infected control mice. Both Hmgcr and Ldlr were in significantly higher abundance at 4 DPI (log_2_ fold changes [log_2_FC], 1.53 and 1.11, respectively), with a more exaggerated increase at 6 DPI (log_2_FC, 2.04 and1.33, respectively). Uniquely, in parallel with the increased abundance of proteins involved in cholesterol biogenesis and import, we found an increase in the cholesterol efflux transporters Abca1 and Abcg5, with a significant increase in Abca1 protein levels at both 4 DPI and 6 DPI (log_2_FC, 1.45 and 2.89, respectively) (Fig. 2B).

10.1128/mBio.00062-19.5 FIG S5 (A) Fold changes in protein levels from levels in mock-infected samples from the same biological experiment involved in the cholesterol biosynthesis pathway. Data were determined by proteomic analysis (*n* = 3). (B) Abca1 expression levels remain unchanged at 3 DPI compared to those in mock-infected samples. Data were determined by qRT-PCR analysis (*n* = 5). Download FIG S5, TIF file, 0.9 MB. Copyright © 2019 Hopkins et al. 2019 Hopkins et al. This content is distributed under the terms of the Creative Commons Attribution 4.0 International license.

While expression of *Hmgcr*, *Ldlr*, and *Pcsk9* is regulated by the transcription factor SREBP2, expression of Abca1, Abcg5, and the inducible degrader of the low-density lipoprotein (LDL) receptor (Idol) is regulated by liver X receptor (LXR), transcription factors which are considered to be mutually antagonistic (23). In order to determine the temporal activation of SREBP2 and LXR, we profiled the expression of *Ldlr*, *Pcsk9*, *Hmgcr*, *Abca1*, *Abcg5*, and *Idol* by quantitative reverse transcription-PCR (qRT-PCR). Unexpectedly, unlike with the proteomics data, this revealed that expression of *Hmgcr* was significantly downregulated at 6 DPI (Fig. 2C), with no significant change at 4 DPI. Moreover, no significant change in the expression of *Ldlr* or *Pcsk9* was seen at either expansion phase time points compared to their expression in mock-infected mice, yet a significant increase in the expression of *Hmgcr*, *Ldlr*, and *Pcsk9* was detected at 8 DPI (Fig. 2D and E). This apparent discrepancy between the proteome and transcriptome at 4 and 6 DPI may reflect changes to the degradation pathway of these proteins during infection or suggest that there is a store of these mRNAs in P bodies. In contrast, expression of *Abca1* showed a temporal increase during the course of infection, with a tendency toward upregulation on 4 DPI, which became significant on 6 DPI and continued to increase on 8 DPI (Fig. 2F). The pattern of *Abca1* expression correlated with what was observed in the quantitative proteomics analysis. In control experiments, no significant increase in the expression of *Abca1* was detected on 3 DPI compared to its expression in mock-infected mice (Fig. S5B). Unexpectedly, transcriptional data of *Abcg5* and *Idol* exhibit significant downregulation at 6 DPI (Fig. 2G and H), which anticorrelates with the corresponding data for *Abca1*, despite reports that all three proteins are under regulation of LXR in the liver. These data suggest either a differential mode of cholesterol regulation in IECs during stress conditions or that C. rodentium has the ability to directly disrupt aspects of cholesterol homeostasis.

### C. rodentium infection induces dysbiosis of colon-associated microbiota at 4 DPI.

**FIG 3:**
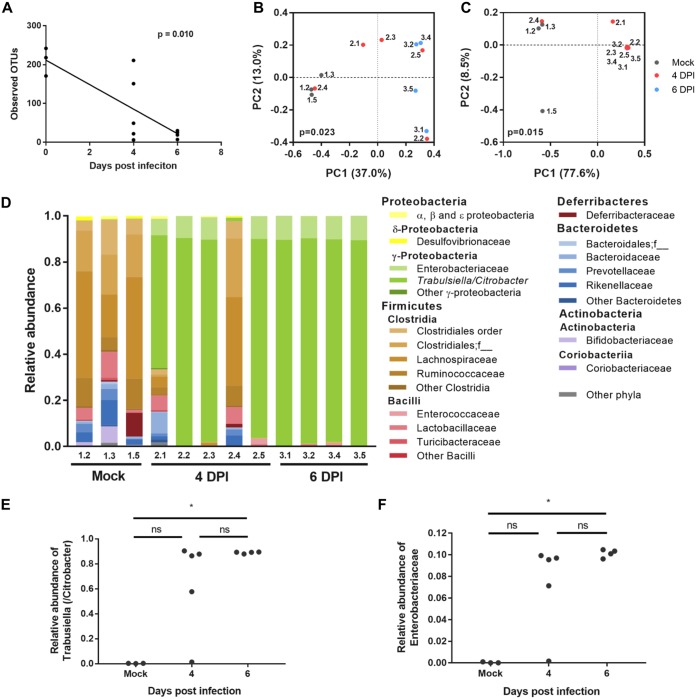
C. rodentium induces colonic mucosal dysbiosis by 4 DPI. Shown are the results of a16S rRNA gene analysis of the distal colonic mucosal microbiota of mock-infected mice and at 4 and 6 DPI. (A) Alpha diversity (observed OTUs), showing a significant decrease over time, as analyzed with the Spearman correlation; (B and C) Jaccard (B) and Bray-Curtis (C) PCoA showing a significant difference in community compositions, analyzed by PERMANOVA; (D) taxonomic compositions of individual mice; (E and F) relative abundances of species of the *Citrobacter/Trabulsiella* genus (E) and Enterobacteriaceae family (not further classified) (F). PC1 and PC2, principal components 1 and 2; *, *P* < 0.05 by the Kruskal-Wallis test with Dunn’s multiple-comparison test. In all panels, a dot or bar represents an individual mouse.

As we found dramatic changes in the metabolic landscape of the epithelium, we next analyzed whether this affected the composition of the mucosa-associated colonic microbiome. This revealed a significant decrease in alpha diversity at both 4 and 6 DPI compared to that in the control mice (Fig. 3A). Weighted and unweighted principal-coordinate analysis (PCoA) showed significant differences in microbiota compositions between the conditions, with mock-infected and 6 DPI mice clustering separately and 4 DPI mice split between the two groups (Fig. 3B and C), which is consistent with the observed mouse-to-mouse variation in CFU counts at 4 DPI (Fig. S6A). Taxonomic analysis showed that C. rodentium (classified by the Greengenes database as *Trabulsiella* [6]) expanded to over 85% of the mucosal bacterial population in 3 out of 5 mice at 4 DPI and in all 4 mice at 6 DPI (Fig. 3D and E). Conversely, analysis of the fecal microbiome in mock-infected and 4 and 8 DPI samples showed no significant differences in observed operational taxonomic units (OTUs) (Fig. S6B), and despite a significant increase in the relative abundances of C. rodentium between mock-infected and 8 DPI mice (Fig. S6D), there was no overall significant change to community composition (Fig. S6C and D). We previously reported that members of the Enterobacteriaceae family, undetectable on the colonic mucosa in mock-infected mice, bloom by 8 DPI (6). Importantly, we observed a bloom in “Enterobacteriaceae” (not further classified) in 4 out of 5 mice as early as the day of colonic colonization (4 DPI) (Fig. 3F). No significant increase in Enterobacteriaceae was observed in the feces (Fig. S6G), suggesting that these observed changes are specific to mucosa-associated commensals, and parallel changes do not occur in the luminal microbiome. Taken together, these results demonstrate that colonic dysbiosis during C. rodentium infection is localized to the mucosa and occurs as early as 4 DPI, coinciding with low-level C. rodentium colonization of the colon and changes to the IECs’ metabolic (e.g., cholesterol biogenesis and efflux) and bioenergetic (e.g., aerobic glycolysis and downregulation of the TCA cycle and OXPHOS) activities.

10.1128/mBio.00062-19.6 FIG S6 (A) CFU-per-gram stool counts. 16S rRNA gene analysis of the stool microbiotas of uninfected mice and at 4 and 8 days after C. rodentium infection. (B) Alpha diversity (observed OTUs) showing no significant decrease overtime, as analyzed with the Spearman correlation. (C and D) Jaccard (C) and Bray-Curtis (D) PCoA analysis showing no significant difference in community composition, analyzed by PERMANOVA. (E) Taxonomic composition of individual mice. (F and G) Relative abundances of species of the *Citrobacter/Trabulsiella* genus (F) and Enterobacteriaceae family (not further classified) (G). **, *P* < 0.05 by the Kruskal-Wallis test with Dunn’s multiple-comparison test. In all panels, a dot or bar represents an individual mouse. Download FIG S6, TIF file, 2.8 MB. Copyright © 2019 Hopkins et al. 2019 Hopkins et al. This content is distributed under the terms of the Creative Commons Attribution 4.0 International license.

### IECs respond to IL-22 from 4 DPI.

IL-22 plays a key role in maintaining the epithelial barrier and preventing C. rodentium dissemination from the gut. Protection of the mucosal surface is achieved via IL-22 signaling to the IECs, which respond by phosphorylation of STAT3, cell proliferation, expression of AMPs, and induction of nutritional immunity (24). Consistently, STAT3 (enrichment score, 0.69; Benjamini-Hochberg FDR, 6.84E–03) and a regulator of STAT3, Enhancer of zeste homolog 2 (EZH2) (25) (enrichment score, 0.54; Benjamini-Hochberg FDR, 3.89E–07) (Fig. S4), as well as E2F1 (enrichment score, 0.69; Benjamini-Hochberg FDR, 0.001) and MYC (enrichment score, 0.20; Benjamini-Hochberg FDR; 7.08E–18), involved in cell cycle regulation and growth, were predicted to be activated as early as the day of colonic colonization (4 DPI) (Fig. S4).

**FIG 4:**
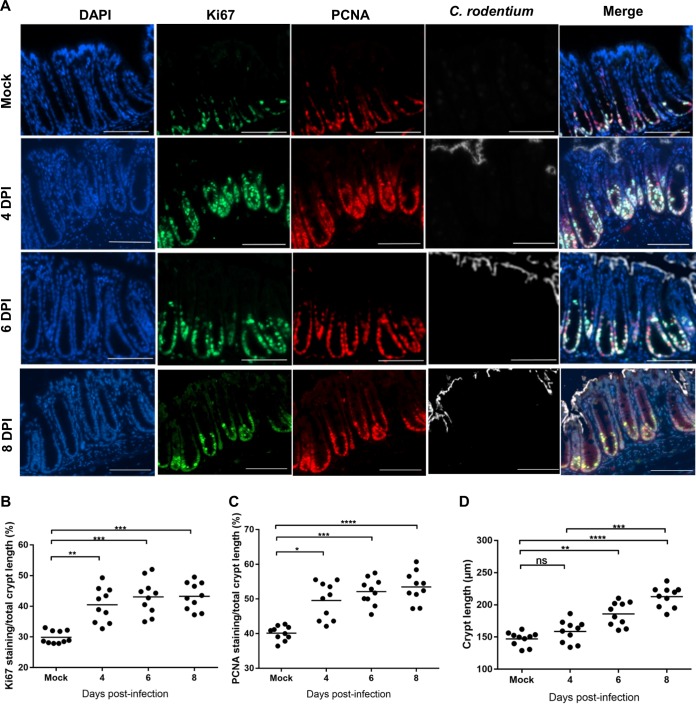
Cell proliferation is initiated before crypt hyperplasia. (A) Representative immunostaining of Ki67 (green), PCNA (red), C. rodentium (white), and DNA (blue) in colon sections from mice mock infected or infected with C. rodentium at 4, 6, or 8 DPI, as indicated (scale bar = 200 µm). (B) Crypt lengths measured from H&E-stained colon sections. (C and D) Fractions of Ki67 (C) or PCNA (D) staining per total crypt length. Each dot represents the mean percentage per mouse (*n* = 10). Significance was determined by Tukey’s multiple-comparison test. *, *P* < 0.05; **, *P* < 0.01; ***, *P* < 0.001; ****, *P* < 0.0001; ns, not significant.

We next analyzed temporal changes to cell proliferation in response to C. rodentium infection by staining colonic sections for the proliferation marker Ki67 and the proliferating cell nuclear antigen (PCNA) (Fig. 4A). This revealed significantly increased staining for both proliferation markers at 4, 6, and 8 DPI compared to that for mock-infected mice (Fig. 4B and C). In contrast, no increase in Ki67 staining was detected at 3 DPI (Fig. S7A and B). Importantly, despite the elevated levels of proliferation markers, no significant increase in crypt length was observed at the day of colonic colonization (4 DPI) (Fig. 4D), suggesting that while the activation of cell proliferation pathways occurs as early as 4 DPI, there is a delay between assembly of the cell proliferation machinery and development of CCH.

10.1128/mBio.00062-19.7 FIG S7 (A) Representative images of immunostaining at 3 DPI of Ki67 (green), PCNA (red), C. rodentium (white), and DNA (blue) in colon sections from mice (*n* = 5) infected with C. rodentium (scale bar = 200 µm). (B) Percentage of Ki67 of total crypt length. Each dot represents the mean percentage per mouse (*n* = 5). Significance was determined by Tukey’s multiple-comparison test. ns, not significant. Download FIG S7, TIF file, 1.5 MB. Copyright © 2019 Hopkins et al. 2019 Hopkins et al. This content is distributed under the terms of the Creative Commons Attribution 4.0 International license.

**FIG 5:**
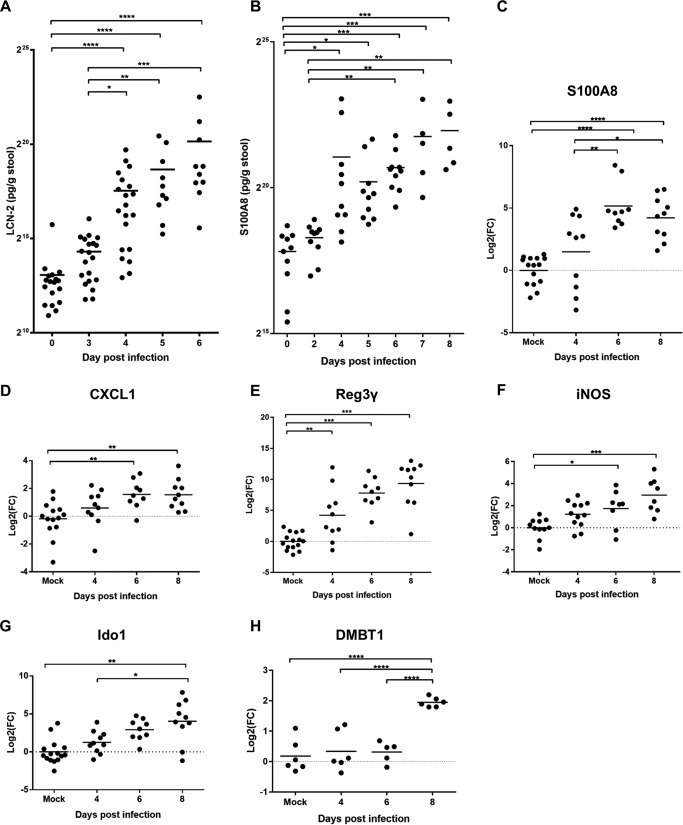
Progression of IL-22 responses during the course of *in vivo* infection. LCN-2 (A) and S100A8 (B) measurements were determined by ELISA on homogenized stool samples collected at frequent intervals from the day of inoculation to a minimum of 6 DPI. Each dot represents a single mouse. Significance was determined using the Kruskal-Wallis multiple-comparison test. qRT-PCR analysis of S100A8 (C), CXCL-1 (D), Reg3γ (E), Ido1 (F), iNOS (G), and DMBT1 (H) expression levels in IECs enriched from mock-infected or infected colons at the time points indicated on the *x* axis. The data are shown as log_2_ fold changes from the average expression level in uninfected mice from the same biological experiment. Statistical significance was determined by Tukey’s multiple-comparison test. *, *P* < 0.05; **, *P* < 0.01; ***, *P* < 0.001; ****, *P* < 0.0001.

We then monitored IL-22-mediated nutritional immunity responses in a temporal fashion by measuring LCN-2 and S100A8 levels in feces from individual C. rodentium-infected mice by ELISA. LCN-2 and S100A8 levels increased significantly from baseline levels by 4 DPI and continue to increase up to 8 DPI (Fig. 5A and B). To further validate these findings, we used qRT-PCR to investigate the level of S100A8 expression in IECs (Fig. 5C). Interestingly, significant induction of S100A8 expression was seen only from 6 DPI in the qRT-PCR data, suggesting that recruited neutrophils might contribute to the overall S100A8 levels observed in feces at 4 DPI, supported by data showing that significant numbers of neutrophils are recruited to the epithelium at 4 DPI (26). Consistent with previous reports (27), expression of the neutrophil chemoattractant CXCL1 in IECs was significantly induced by 6 DPI, with an increasing trend observed at 4 DPI compared to the trend in mock-infected mice (Fig. 5D). However, the neutrophil chemoattractant CXCL5 was found significantly upregulated at 4 DPI in the proteomics data (Fig. 1F). In control experiments, no expression of S100A8 or CXCL1 was detected on 3 DPI (Fig. S8A and B).

10.1128/mBio.00062-19.8 FIG S8 S100A8 (A), CXCL1 (B), and Reg3 expression levels remained unchanged at 3 DPI compared to levels in mock-infected samples. Determined by qRT-PCR analysis. Download FIG S8, TIF file, 0.9 MB. Copyright © 2019 Hopkins et al. 2019 Hopkins et al. This content is distributed under the terms of the Creative Commons Attribution 4.0 International license.

We used Reg3γ and iNOS, responsible for producing reactive nitrogen intermediates, as reporters for IL-22-induced expression of AMPs (28, 29). Employing qRT-PCR, we observed that Reg3γ expression was significantly induced in IECs by the day of colonic colonization (4 DPI) (Fig. 5E) and continued to increase to the peak of infection. Furthermore, our results revealed an increasing trend in *iNOS* expression during C. rodentium infection which was statistically significant at 6 DPI and 8 DPI (Fig. 5F). These results suggest that despite IL-22 levels peaking at 4 DPI (18), induction of some IL-22-mediated genes is significantly induced by this time point and then continues to increase during infection to 8 DPI. On the other hand, DMBT1, a glycoprotein that has been shown to inhibit bacterial attachment to IECs (30) and Ido1 (indoleamine 2,3-dioxygenase 1), a marker for IFN-γ activity, were significantly induced only at 8 DPI (Fig. 5G and H). In control experiments, no significant increase in expression of *Reg3γ* or *iNOS* was detected on 3 DPI (Fig. S8C and D).

### C. rodentium reprograms the composition of crypt cell populations.

**FIG 6:**
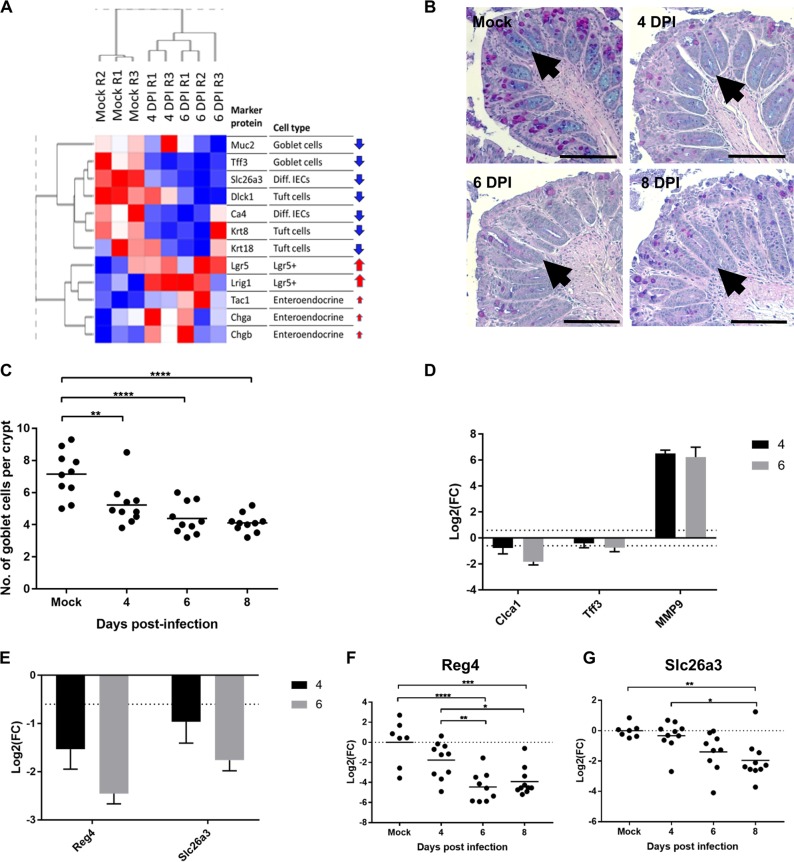
C. rodentium infection induces reprogramming of the epithelium. (A) Intestinal cell type signatures during progression of infection. Shown are relative expression levels (normalized abundances) of proteins (rows) across samples (columns). Red arrows indicate an observed increasing trend in protein level as infection progresses, while blue arrows indicate a decrease. Magnitude of change is reflected by the arrow size. Diff., differentiated. (B) Representative images of AB/PAS staining of mouse colonic section from the distal end. (C) Number of goblet cells per colonic crypt from colonic sections stained with AB/PAS. Each data point represents the mean number of goblet cells for a single mouse (*n* = 10). Significance was determined by Tukey’s multiple-comparison test. *, *P* < 0.05; **, *P* < 0.01; ***, *P* < 0.001; ****, *P* < 0.0001. (D) Log_2_ fold changes at either 4 DPI or 6 DPI, as indicated, compared to levels in mock-infected mice calculated from the proteomics data set for the goblet cell markers Clca1, Tff3, and MMP9. (E) Levels of Reg4 and Slc26a3 (differentiated epithelial cell markers as quantified by proteomics). (F and G) Validation by qRT-PCR of Reg4 (F) and Slc26a3 (G) expression levels.

IFN-γ plays a role in goblet cell depletion, which itself protects mice from C. rodentium infection (31). We therefore analyzed the effect of C. rodentium infection on the compositions of different crypt cell populations over time. Hierarchical clustering and analysis of protein biomarkers for different epithelial cell types revealed a temporal decrease in goblet cells, tuft cells, and differentiated IECs during C. rodentium infection (Fig. 6A). To validate the kinetics of goblet cell depletion, Alcian Blue-periodic acid-Schiff (AB/PAS) staining, which stains all intracellular mucin glycoproteins, was used to identify and subsequently count goblet cells in mouse distal colon sections (Fig. 6B). A significant decrease in the average number of goblet cells per crypt per mouse was observed on the day of colonic colonization (4 DPI), in addition to 6 and 8 DPI, compared to the number in the mock control (Fig. 6C). Consistently, the goblet cell marker protein Clca1 was found to be in lower abundance in the proteome at both 4 and 6 DPI, while the marker Tff3 was found to be in lower abundance at 6 DPI (Fig. 6D). Notably, MMP9, a negative regulator of terminal differentiation of goblet cells in the colon, ranked as the 5th-most-upregulated protein at 6 DPI (Fig. 1F), was also in significantly higher abundance at 4 DPI than in mock-infected controls (Fig. 6D). In addition to changes in goblet cell abundance during infection, there was a decrease in Reg4, a marker for DCS cells from 6 DPI, shown by proteomics and qRT-PCR analyses (Fig. 6E and F). Slc26a3, a marker for terminally differentiated enterocytes, was also found in lower abundance at 4 and 6 DPI in the proteomic data set (Fig. 6E), although transcripts were significantly lower only on 8 DPI (Fig. 6G). Consistently, the transcription factor peroxisome proliferator-activated receptor gamma (PPARG), which is involved in promoting the expression of genes involved in differentiation, was predicted to be significantly inactivated (enrichment score, −0.51; Benjamini-Hochberg FDR, 2.75E–02) (Fig. S4).

Taken together, these results suggest that while C. rodentium colonizes the top of the crypts at a low level during the expansion phase of the infection (from the day of colonic colonization), it temporally reshapes the architecture and cellular composition all the way down to the stem cell niche, starting with depletion of goblet cells and then DCS cells and, finally, terminally differentiated enterocytes.

## DISCUSSION

Proteomic analysis has been extensively used to study intracellular pathogen-host interactions during infections (32–35); however, fewer such studies have been conducted on extracellular bacterial infections (36, 37). Furthermore, exploring temporal changes during *in vivo* bacterial infection at the protein level is relatively unexplored, with most temporal proteomics studies focusing on viral infection of cell culture, for example, hepatitis C virus (HCV) infection (38). Using an isobaric-labeling approach to map proteome-wide changes during C. rodentium infection of C57BL/6 mice, we have been able to quantify 9,127 mouse proteins from IECs and to assess significant changes in abundance during the expansion phase of infection. Further to validating what has been observed previously at the steady-state phase of C. rodentium infection (6, 39), we have increased the temporal resolution of this approach and have, unexpectedly, discovered a rapid onset of changes in IECs occurring immediately upon migration of bacteria into the colon. Changes to the metabolic profile of the IECs coincided with the depletion of goblet cells and expansion of Enterobacteriaceae on the day of colonic colonization (4 DPI).

Surprisingly, despite low levels of shedding and sporadic adhesion at 4 DPI, we observed significant perturbations in the proteome, including the establishment of an inverse correlation between repression of central metabolic pathways and upregulation of processes involved in DNA damage repair and replication. Notably, previous proteomics work revealed similar patterns of upregulation of DNA replication, mismatch repair, and cell cycle, concomitantly with downregulation of steroid hormone biosynthesis, metabolic pathways, the PPAR signaling pathway, the TCA cycle, and OXPHOS within a group of cell lines categorized as the colorectal proteomic subtype 1 (40). This group of colorectal cell lines include a wide range of cell types, including stem, goblet, inflammatory, and TA cells (40–42). Given that this anticorrelation of processes can be detected within a single cell line and cell lines which correspond with different cell types, this suggests that the polarization that we observe during *in vivo* infection likely occurs within the same cells, as opposed to some processes being upregulated in one cell type and others being downregulated in another. Furthermore, the establishment of the inverse-correlation state between proliferation and metabolism is detected as early as the day of colonic colonization (4 DPI), when pathogen adhesion is sporadic; thus, polarization may be independent of direct bacterial contact and might represent a global epithelial response to diffusible signals, such as cytokines, or direct cell-to-cell communication between IECs.

Recent reports suggest involvement of cholesterol in innate immune responses (43). Furthermore, cholesterol biogenesis was upregulated during C. rodentium infection (6), resulting in increased serum cholesterol (44). Here, we observed an increased abundance of Abca1 by 4 DPI in the proteomics data and significant transcriptional induction by 6 DPI. Importantly, other LXR-regulated genes, *Abcg5* and *Idol*, were not transcriptionally induced and yet were found in higher abundance in the proteomes of infected IECs than in controls. Similarly, transcription of SREBP2-regulated genes (*Hmgcr*, *Pcsk9*, and *Ldlr*) was not induced until 8 DPI, although the corresponding proteins were found in higher abundance in infected IECs than in controls. These results suggest that LXR and SREBP2 regulation of their target genes in IECs is somewhat different from their well-studied activities in the liver, the major site where the bodily cholesterol homeostasis is controlled (23). Furthermore, these data show that as early as 4 DPI, both cholesterol biogenesis and efflux proteins are simultaneously in higher abundance in IECs. However, we still need to determine directionality in terms of cholesterol control; i.e., it is not apparent if accumulation of hydroxycholesterol due to cholesterol biogenesis triggers LXR activation or whether efflux, via Abca1 and Abcg5/8, leads to cholesterol depletion and activation of SREBP2.

Genera of the Enterobacteriaceae family, such as *Serratia*, *Dickeya*, and *Erwinia*, are able to metabolize cholesterol (45, 46). Consistently, Enterobacteriaceae were found to bloom on the mucosal surface at 4 DPI concomitantly with upregulation of host cholesterol biosynthesis and export. Downregulation of β-oxidation at 4 DPI, which increases mucosal oxygenation, likely also favors the expansion of facultative anaerobes at the expense of obligate anaerobes. Although quantification of C. rodentium CFU in stool samples showed a 10-fold increase between 4 and 6 DPI (Fig. 1A), microbiome analysis showed that C. rodentium represents up to 80 to 90% of the mucosa-associated bacteria in the majority of mice at 4 DPI (Fig. 3E). Thus, we find that changes to the gut environment have already excluded the majority of the normal mucosal flora as early as the day of colonic colonization (4 DPI). Importantly, however, dysbiosis was localized to the mucosa, with no significant changes in luminal microbiota community structure (see Fig. S6 in the supplemental material). These results suggest that changes to metabolism in IECs, such as the switch from aerobic respiration to the Warburg effect, facilitate the expansion of Enterobacteriaceae, which might cooperate with the innate immune response in fighting C. rodentium.

We also observed clear IL-22 responses at 4 DPI, illustrated by induced expression of AMPs (e.g., Reg3γ) and nutritional immunity (e.g., LCN-2). However, while Tsai and colleagues have shown upregulation of Claudin-2 at 2 DPI (20), which was found in higher abundance in the 4 DPI proteome than at other times, none of the responses measured in this study were induced at 3 DPI, correlating with the absence of visible C. rodentium in the colon at this time point. This suggests that the alarm in IECs is not raised during the establishment phase (when C. rodentium resides in the cecum) but instead as soon as the colon is seeded with C. rodentium at 4 DPI. On the other hand, both proteomic and transcriptional profiling of Ido1 and iNOS show no statistically significant increase in levels at 4 DPI compared to levels in mock-infected controls, with iNOS being significantly induced by 6 DPI in proteomics (log_2_FC, 2.40) and qRT-PCR data (Fig. 5G) and Ido1 being significantly induced at 8 DPI (Fig. 5F). These data suggest that an IFN-γ response is significantly induced only at the late expansion phase, when bacterial load is higher, rather than on the first day of colonization (4 DPI).

Importantly, although we observed significant expansion of the Ki67-positive zone, as well as induction of Slc5A9 (which feeds aerobic glycolysis), at the day of colonic colonization (4 DPI), no significant CCH was detected at this time point. Taken together, these results suggest that sensing the presence of a pathogen triggers robust colonic responses, which might potentially be a preparatory mechanism in case of pathogenic expansion. Indeed, early proliferation and STAT3 activation have recently been seen in the small intestine following Listeria monocytogenes mouse infection (47).

It has previously been shown that changes in the cell type populations of the epithelium occur during C. rodentium infection, with significant reduction of mucin-containing goblet cells being observed at the peak of infection (1, 8). Here, using a combination of proteomic, histology, and mRNA expression techniques, we found that reprogramming of the epithelial layer also occurs as early as 4 DPI, which includes significant depletion of goblet cells, differentiated epithelial cells, tuft cells, and DCS cells, with an upregulation in LGR5^+^ cells (Fig. 6A). Importantly, these changes occur in the absence of significant crypt hyperplasia (Fig. 4D). The decreased abundance of Reg4 and Slc26a3, markers of DCS and differentiated epithelial cells, respectively, was observed as soon as 4 DPI. Consistently, DCS provides Notch signals to the stem cell niche, which is critical to terminal cell differentiation (2). However, transcriptionally, a decline in these markers was seen only at 6 DPI (Notch signals) and 8 DPI (stem cells), suggesting that protein degradation precedes mRNA turnover. Loss of goblet cells results in a thinner mucus layer, which has been suggested to coincide with a reduction in nutrient availability for C. rodentium, supported by a study where goblet cell numbers were increased with dibenzazepine and resulted in impaired host defense (31). Moreover, depletion of goblet cells might provide the commensal Enterobacteriaceae access to the surface of IECs, enabling them to expand in a novel niche.

This study has revealed that the early signatures of colonic C. rodentium infection are reprogramming the metabolic and physiological state of the mucosal surface. Depletion of goblet cells reduces the amount of mucus, which alongside changes to metabolism and oxygen availability, allows accessibility of Gram-negative commensals to the mucosa, followed by expansion, which might assist the host in expelling the pathogen. Accordingly, we report for the first time that, parallel to innate immune responses, there are inherent IEC and microbiome responses to infection at the onset of mouse colonic infection. Considering that C. rodentium, EPEC, and EHEC share an infection strategy and virulence factors, the intestinal responses in mice infected with C. rodentium likely reflect human responses to infection with extracellular pathogens.

## MATERIALS AND METHODS

### Bacterial strains.

Citrobacter rodentium strain ICC169 (7, 48) was grown in Miller lysogeny broth (LB; Invitrogen) or on LB solidified with 3.7% agar, supplemented with ampicillin (100 μg/ml), kanamycin (50 μg/ml), nalidixic acid (NAL) (30 μg/ml), or gentamicin (Gm) (10 mg/ml), as required, and grown overnight at 37°C with shaking at 200 rpm (liquid cultures) or stationary (for plates).

### Animals procedures.

All animal experiments were performed in accordance with the Animals Scientific Procedures Act of 1986 and were approved by the local Ethical Review Committee and UK Home office guidelines. Experiments were designed in agreement with the ARRIVE guidelines (49) for the reporting and execution of animal experiments. Specific-pathogen-free female C57BL/6 mice (18 to 20 g) were purchased from Charles River, London, United Kingdom. All mice were housed in individually HEPA-filtered cages with sterile bedding (processed corncobs, grade 6), nesting (LBS Serving technology), and free access to sterilized food (LBS Serving technology) and water. For each experiment, 5 mice were randomly assigned to each group.

### Oral gavage of mice and CFU count.

Overnight cultures of C. rodentium strain ICC169 were pelleted and subsequently concentrated 10-fold in sterile phosphate-buffered saline (PBS) (∼5 × 10^9^ CFU/ml).

Mice were inoculated by oral gavage with 200 μl of the concentrated inoculum, and the number of viable bacteria used as inoculum was determined by retrospective plating onto LB agar containing NAL. Mock-treated mice were orally gavaged with 200 μl PBS. Stool samples were collected daily after inoculation, and the number of viable bacteria per gram of stool was determined by serial dilution and plating onto LB agar containing NAL.

### Extraction of enterocytes.

At 4 or 6 DPI, a 4-cm segment of terminal colon was cut longitudinally, placed in 4 ml enterocyte dissociation buffer (1× Hanks’ balanced salt solution without Mg and Ca, containing 10 mM HEPES, 1 mM EDTA, and 5 ml/ml 2-β-mercaptoethanol), and incubated at 37°C with shaking for 45 min. The remaining tissue was removed and stored at −80°C, and lifted enterocytes were collected by centrifugation (2,000 × *g* for 10 min), followed by two PBS washes. Enterocyte pellets were stored at −80°C for either proteomics or qRT-PCR experiments.

### Histological analysis.

The distal 0.5 cm of the colon was fixed in 4% paraformaldehyde (PFA) for 2.5 h and then submersed in 70% ethanol. Fixed tissues were processed, paraffin embedded, and sectioned at 5 μm by Lorraine Lawrence (histology facility, Imperial College London). Formalin-fixed, paraffin-embedded (FFPE) sections were then stained with either hematoxylin and eosin (H&E) or Alcian blue and periodic acid-Schiff (AB/PAS) stain, using standard techniques, or processed for immunofluorescence.

H&E-stained sections were analyzed using a 20× lens objective on the Zeiss Axio Observer Z1 microscope. At least 20 well-oriented crypts from each section from each individual mouse were measured, and an average crypt length for each mouse was calculated, using ZEN 2.3 Lite software (Zeiss). Similarly, Ki67 staining was also measured by this method and divided by the total length of the crypt to calculate the percentage of Ki67 staining. The same protocol was used to analyze PCNA staining in the crypt. The density of goblet cells was calculated by counting the number of goblet cells per crypt and measuring the total crypt length.

FFPE sections were dewaxed by submersing them in Histo-Clear solution twice for 10 min, 100% ethanol (EtOH) twice for 10 min, 95% EtOH twice for 3 min, 80% EtOH once for 3 min, and PBS-0.1% Tween 20-0.1% saponin (PBS-TS) twice for 3 min. Subsequently, sections were then heated for 20 min in demasking solution (0.3% trisodium citrate-0.05% Tween 20 in distilled H_2_O). Once cooled, slides were first blocked in PBS-TS supplemented with 10% normal donkey serum (NDS) for 20 min in a humid chamber, before being incubated with primary antibody diluted in PBS-TS with 10% NDS for 1 h. Ki67 (Abcam) was diluted 1:50, PCNA (Abcam) was diluted 1:500, and intimin 280B against porcine EPEC (50) was diluted at 1:50. Slides were rinsed twice for 10 min each time in PBS-TS, followed by incubation with secondary antibodies, DAPI (4′,6-diamidino-2-phenylindole), and phalloidin, which was again diluted in PBS-TS supplemented with NDS for 1 h. All secondary antibodies (Jackson Immunoresearch) were used at 1:100, while the reagents DAPI (Thermo Fisher) and phalloidin-iFluor 647 conjugate (Thermo Fisher) were used at 1:1,000 and 1:100, respectively. Washing steps were repeated before slides were mounted with ProLong Gold antifade mountant (Thermo Fisher Scientific). Slides were imaged the subsequent day using a Zeiss Axio Observer Z1 microscope with a 20× lens objective. Images were acquired with AxioVision 4.3 software and subsequently analyzed using ImageJ (Fiji).

### LCN-2 and S100A8 ELISA.

Stool samples were homogenized in PBS with 0.1% Tween 20 (PBST) using a vortex machine for 15 min. Samples were centrifuged at 16,000 rpm for 10 min, and the supernatant was extracted and stored at −80°C. LCN-2 concentration was determined using a DuoSet mouse lipocalin-2 ELISA or DuoSet mouse S100A8 ELISA (R&D Systems), according to the manufacturer’s instructions.

### Quantitative real-time RT-PCR.

RNA from enriched IECs was isolated with an RNeasy minikit (Qiagen) by following the manufacturer’s instructions. RNA was treated with RQ1 RNase-free DNase (Promega), and subsequently, cDNA was synthesized using a Moloney murine leukemia virus reverse transcription kit (Promega). Quantitative RT-PCR was performed using Fast SYBR green master mix (Thermo Fischer Scientific) and different primer pairs (see Table S2 in the supplemental material).

10.1128/mBio.00062-19.10 TABLE S2 Primer sequences. Download Table S2, PDF file, 0.01 MB. Copyright © 2019 Hopkins et al. 2019 Hopkins et al. This content is distributed under the terms of the Creative Commons Attribution 4.0 International license.

### Sample preparation for TMT labeling.

Both IEC pellets isolated from mice and CMT-93 cell pellets were dissolved in 150 μl 0.1 M triethylammonium bicarbonate (TEAB), 0.1% SDS, 10% isopropanol on ice, assisted with pulsed probe sonication. Samples were subsequently boiled at 90°C for 5 min on a shaking device at 300 rpm. Protein concentration was measured with the Quick Start Bradford protein assay (Bio-Rad) according to the manufacturer’s instructions. Aliquots containing 100 μg of total protein, with an equal contribution from each individual mouse within the group, were prepared for trypsin digestion. Samples were reduced with 5 mM tris-2-carboxyethyl phosphine (TCEP) and alkylated with 10 mM iodoacetamide (IAA). Proteins were then digested by adding 75 ng/μl trypsin to each sample and incubating the samples overnight. The resultant peptides were diluted up to 100 μl with 0.1 M TEAB buffer and labeled with a tandem mass tag (TMT) multiplex reagent vial (Thermo Scientific) according to the manufacturer’s instructions. Hydroxylamine was used to quench the reaction, and then all 11 samples were combined in equal amounts to a single tube. The sample was then dried with a centrifugal vacuum concentrator.

### Basic reverse-phase peptide fractionation and LC-MS/MS analysis.

Offline high-pH reverse-phase (RP) peptide fractionation was performed using the Waters XBridge C_18_ column (2.1 by 150 mm, 3.5 mm) on a Dionex Ultimate 3000 high-performance liquid chromatograph (HPLC) system. Mobile phase A was 0.1% ammonium hydroxide, and mobile phase B was 100% acetonitrile, 0.1% ammonium hydroxide. The TMT-labeled peptide mixture was reconstituted in 100 ml mobile phase A and was fractionated using a multistep gradient elution method at 0.2 ml/min as follows: isocratic for 5 min at 5% phase B, gradient for 35 min to 35% phase B, gradient to 80% phase B in 5 min, isocratic for 5 min, and reequilibrated to 5% phase B. Fractions were collected every 30 s and vacuum dried. LC-MS/MS analysis was performed on the Dionex Ultimate 3000 system coupled with the Orbitrap Fusion mass spectrometer (Thermo Scientific). Each peptide fraction was reconstituted in 40 μl 0.1% formic acid, and 7 μl was loaded to the Acclaim PepMap 100, 100-μm by 2-cm, 5-μm, 100-Å C_18_ trapping column at a 10-μl/min flow rate. The sample was then subjected to a gradient elution on the Acclaim PepMap rapid-separation LC (75 μm by 50 cm, 2 μm, 100 Å) C_18_ capillary column at 45°C. Mobile phase A was 100% H_2_O, 0.1% formic acid, and mobile phase B was composed of 80% acetonitrile, 0.1% formic acid. The gradient separation method at a flow rate of 300 nl/min was as follows: gradient for 90 min from 10% to 38% phase B and for 10 min up to 95% phase B, isocratic for 5 min at 95% B, reequilibrated to 5% phase B in 5 min, and isocratic for 10 min at 10% phase B. Precursors between 375 and 1,500 *m/z* were selected, with mass resolution of 120,000, automatic gain control (AGC) of 4 × 10^5^, and IT (injection time) of 50 ms, with the top speed mode in 3 s, and the precursors were isolated for collision-induced dissociation (CID) fragmentation with a quadrupole isolation width of 0.7 Th (Thomson unit). Collision energy was set at 35%, with AGC at 1 × 10^4^ and IT at 50 ms. MS3 quantification was obtained with higher-energy collisional dissociation (HCD) fragmentation of the top 7 most abundant CID fragments isolated with synchronous precursor selection (SPS). Quadrupole isolation width was set at 0.7 Th, collision energy was applied at 65%, and the AGC setting was at 1 × 10^5^ with IT at 105 ms. The HCD MS3 spectra were acquired for the mass range 100 to 500 with a resolution of 50,000. Targeted precursors were dynamically excluded for further isolation and activation for 45 s with 7-ppm mass tolerance.

### Database search and protein quantification.

The SEQUEST-HT search engine was used to analyze the acquired mass spectra in Proteome Discoverer 2.1 (Thermo Scientific) for protein identification and quantification. The precursor mass tolerance was set at 20 ppm, and the fragment ion mass tolerance was set at 0.5 Da. Spectra were searched for fully tryptic peptides with maximum 2 mis-cleavages. TMT6plex at the N terminus/K and carbamidomethyl at the C terminus were defined as static modifications. Dynamic modifications included oxidation of M and deamidation of N/Q. Peptide confidence was estimated with the Percolator node. The peptide FDR was set at 0.01, and validation was based on the *q* value and a decoy database search. All spectra were searched against UniProt reference proteomes of Mus musculus and C. rodentium protein entries. The reporter ion quantifier node included a TMT 11-plex quantification method with an integration window tolerance of 15 ppm and integration method based on the most confident centroid peak at the MS3 level. Only unique peptides were used for quantification, with protein groups considered for peptide uniqueness. Peptides with an average reported signal-to-noise ratio of >3 were used for protein quantification. The enterocytes obtained from mock-infected mice were used as controls for log_2_ ratio calculations.

### Data analysis.

Differential expression *P* values were calculated using a single-sample *t* test in the Perseus proteomics tool (51). Average log_2_ fold changes were used for duplicate measurements of a single time point. KEGG pathway and GOBP slim pathway enrichment analysis was performed with the 1D-annotation enrichment method (52). Terms with significant positive enrichment were addressed as upregulated and those with negative enrichment as downregulated. All terms were filtered for a Benjamini-Hochberg FDR of <0.05 or an FDR of <0.1.

### 16S rRNA gene sequencing and microbiome analysis.

Upon necroscopy, 1 cm of distal colon was immediately frozen and stored at −80°C. DNA was extracted using a Power Soil kit (MO Bio). The 16S V4 region was amplified using 515F/806R primers and sequenced using 2× 250-bp paired-end sequencing (Illumina MiSeq). Sequences were analyzed using the Qiime2 (Quantitative Insights into Microbial Ecology 2; http://www.qiime2.org) analysis pipeline (53). Briefly, FASTA-quality files and a mapping file indicating the barcode sequence corresponding to each sample were used as inputs. Sample reads were separated according to their barcode. Reads were denoised, low-quality reads were trimmed, and paired reads were assembled into longer reads using the DADA2 plugin (54). Samples with less than 15,000 quality reads were excluded from downstream analysis. Taxonomy was assigned using a naive Bayes classifier trained against the Greengenes database (version 13_8), trimmed to contain only the V4 hypervariable region and preclustered at 99% sequence identity. For beta-diversity analysis, weighted (Bray-Curtis) and unweighted (Jaccard) distance measurements were plotted according to the two principal coordinates, and significance between group distances was analyzed using an all-group permutational multivariate analysis of variance (PERMANOVA).

### Statistical analysis.

Data were analyzed by a paired two-tailed Student *t* test or one-way ANOVA with Tukey’s multiple-comparison posttest, as detailed in the figure legends. PRISM 7 (GraphPad) was used to plot line graphs, bar charts, and dot plots. Volcano plots and distribution plots were drawn in RStudio with the ggplot2 and ggrepel packages.

### Data availability.

The mass spectrometry proteomics data have been deposited in the ProteomeXchange Consortium via the PRIDE partner repository (55) with the data set identifier PXD012031.

## References

[1] Haber AL, Biton M, Rogel N, Herbst RH, Shekhar K, Smillie C, Burgin G, Delorey TM, Howitt MR, Katz Y, Tirosh I, Beyaz S, Dionne D, Zhang M, Raychowdhury R, Garrett WS, Rozenblatt-Rosen O, Shi HN, Yilmaz O, Xavier RJ, Regev A 2017 A single-cell survey of the small intestinal epithelium. Nature 551:333–339. doi:10.1038/nature24489. 29144463 PMC6022292

[2] Sasaki N, Sachs N, Wiebrands K, Ellenbroek SIJ, Fumagalli A, Lyubimova A, Begthel H, van den Born M, van Es JH, Karthaus WR, Li VSW, López-Iglesias C, Peters PJ, van Rheenen J, van Oudenaarden A, Clevers H 2016 Reg4 deep crypt secretory cells function as epithelial niche for Lgr5 stem cells in colon. Proc Natl Acad Sci U S A 113:E5399–E5407. doi:10.1073/pnas.1607327113. 27573849 PMC5027439

[3] Peterson LW, Artis D 2014 Intestinal epithelial cells: regulators of barrier function and immune homeostasis. Nat Rev Immunol 14:141–153. doi:10.1038/nri3608. 24566914

[4] Mundy R, MacDonald TT, Dougan G, Frankel G, Wiles S 2005 *Citrobacter rodentium* of mice and man. Cell Microbiol 7:1697–1706. doi:10.1111/j.1462-5822.2005.00625.x. 16309456

[5] Collins JW, Keeney KM, Crepin VF, Rathinam VK, Fitzgerald K, Finlay BB, Frankel G 2014 *Citrobacter rodentium*: infection, inflammation and the microbiota. Nat Rev Microbiol 12:612–623. doi:10.1038/nrmicro3315. 25088150

[6] Berger CN, Crepin VF, Roumeliotis TI, Wright JC, Carson D, Pevsner-Fischer M, Furniss RCD, Dougan G, Bachash M, Yu L, Clements A, Collins JW, Elinav E, Larrouy-Maumus GJ, Choudhary JS, Frankel G 2017 *Citrobacter rodentium* subverts ATP flux and cholesterol homeostasis in intestinal epithelial cells *in vivo*. Cell Metab 6:738–752.e6. doi:10.1016/j.cmet.2017.09.003. PMC5695859 28988824

[7] Wiles S, Clare S, Harker J, Huett A, Young D, Dougan G, Frankel G 2004 Organ specificity, colonization and clearance dynamics i*n vivo* following oral challenges with the murine pathogen *Citrobacter rodentium*. Cell Microbiol 6:963–972. doi:10.1111/j.1462-5822.2004.00414.x. 15339271

[8] Wiles S, Pickard KM, Peng K, MacDonald TT, Frankel G 2006 *In vivo* bioluminescence imaging of the murine pathogen *Citrobacter rodentium*. Infect Immun 74:5391–5396. doi:10.1128/IAI.00848-06. 16926434 PMC1594854

[9] Lupp C, Robertson ML, Wickham ME, Sekirov I, Champion OL, Gaynor EC, Finlay BB 2007 Host-mediated inflammation disrupts the intestinal microbiota and promotes the overgrowth of Enterobacteriaceae. Cell Host Microbe 2:119–129. doi:10.1016/j.chom.2007.06.010. 18005726

[10] Pham TAN, Clare S, Goulding D, Arasteh JM, Stares MD, Browne HP, Keane JA, Page AJ, Kumasaka N, Kane L, Mottram L, Harcourt K, Hale C, Arends MJ, Gaffney DJ, Dougan G, Lawley TD 2014 Epithelial IL-22RA1-mediated fucosylation promotes intestinal colonization resistance to an opportunistic pathogen. Cell Host Microbe 16:504–516. doi:10.1016/j.chom.2014.08.017. 25263220 PMC4190086

[11] Wolk K, Sabat R 2006 Interleukin-22: a novel T- and NK-cell derived cytokine that regulates the biology of tissue cells. Cytokine Growth Factor Rev 17:367–380. doi:10.1016/j.cytogfr.2006.09.001. 17030002

[12] Zenewicz LA, Flavell RA 2011 Recent advances in IL-22 biology. Int Immunol 23:159–163. doi:10.1093/intimm/dxr001. 21393631

[13] Rutz S, Eidenschenk C, Ouyang W 2013 IL-22, not simply a Th17 cytokine. Immunol Rev 252:116–132. doi:10.1111/imr.12027. 23405899

[14] Muñoz M, Eidenschenk C, Ota N, Wong K, Lohmann U, Kühl AA, Wang X, Manzanillo P, Li Y, Rutz S, Zheng Y, Diehl L, Kayagaki N, van Lookeren-Campagne M, Liesenfeld O, Heimesaat M, Ouyang W 2015 Interleukin-22 induces interleukin-18 expression from epithelial cells during intestinal infection. Immunity 42:321–331. doi:10.1016/j.immuni.2015.01.011. 25680273

[15] Lee YS, Yang H, Yang JY, Kim Y, Lee SH, Kim JH, Jang YJ, Vallance BA, Kweon MN 2015 Interleukin-1 (IL-1) signaling in intestinal stromal cells controls KC/CXCL1 secretion, which correlates with recruitment of IL-22- secreting neutrophils at early stages of *Citrobacter rodentium* infection. Infect Immun 83:3257–3267. doi:10.1128/IAI.00670-15. 26034212 PMC4496604

[16] Backert I, Koralov SB, Wirtz S, Kitowski V, Billmeier U, Martini E, Hofmann K, Hildner K, Wittkopf N, Brecht K, Waldner M, Rajewsky K, Neurath MF, Becker C, Neufert C 2014 STAT3 Activation in Th17 and Th22 cells controls IL-22-mediated epithelial host defense during infectious colitis. J Immunol 193:3779–3791. doi:10.4049/jimmunol.1303076. 25187663

[17] Guo X, Qiu J, Tu T, Yang X, Deng L, Anders RA, Zhou L, Fu YX 2014 Induction of innate lymphoid cell-derived interleukin-22 by the transcription factor STAT3 mediates protection against intestinal infection. Immunity 40:25–39. doi:10.1016/j.immuni.2013.10.021. 24412612 PMC3919552

[18] Zheng Y, Valdez PA, Danilenko DM, Hu Y, Sa SM, Gong Q, Abbas AR, Modrusan Z, Ghilardi N, De Sauvage FJ, Ouyang W 2008 Interleukin-22 mediates early host defense against attaching and effacing bacterial pathogens. Nat Med 14:282–289. doi:10.1038/nm1720. 18264109

[19] Kebir H, Kreymborg K, Ifergan I, Dodelet-Devillers A, Cayrol R, Bernard M, Giuliani F, Arbour N, Becher B, Prat A 2007 Human TH17 lymphocytes promote blood-brain barrier disruption and central nervous system inflammation. Nat Med 13:1173–1175. doi:10.1038/nm1651. 17828272 PMC5114125

[20] Tsai PY, Zhang B, He WQ, Zha JM, Odenwald MA, Singh G, Tamura A, Shen L, Sailer A, Yeruva S, Kuo WT, Fu YX, Tsukita S, Turner JR 2017 IL-22 upregulates epithelial claudin-2 to drive diarrhea and enteric pathogen clearance. Cell Host Microbe 21:671–681.e4. doi:10.1016/j.chom.2017.05.009. 28618266 PMC5541253

[21] Berger CN, Crepin VF, Roumeliotis TI, Wright JC, Serafini N, Pevsner-Fischer M, Yu L, Elinav E, Di Santo JP, Choudhary JS, Frankel G 2018 The *Citrobacter rodentium* type III secretion system effector EspO affects mucosal damage repair and antimicrobial responses. PLoS Pathog 14:e1007406. doi:10.1371/journal.ppat.1007406. 30365535 PMC6221368

[22] Snoeks L, Weber CR, Turner JR, Bhattacharyya M, Wasland K, Savkovic SD 2008 Tumor suppressor Foxo3a is involved in the regulation of lipopolysaccharide-induced interleukin-8 in intestinal HT-29 cells. Infect Immun 76:4677–4685. doi:10.1128/IAI.00227-08. 18678662 PMC2546851

[23] Spann NJ, Glass CK 2013 Sterols and oxysterols in immune cell function. Nat Immunol 14:893–900. doi:10.1038/ni.2681. 23959186

[24] Pickert G, Neufert C, Leppkes M, Zheng Y, Wittkopf N, Warntjen M, Lehr H-A, Hirth S, Weigmann B, Wirtz S, Ouyang W, Neurath MF, Becker C 2009 STAT3 links IL-22 signaling in intestinal epithelial cells to mucosal wound healing. J Exp Med 206:1465–1472. doi:10.1084/jem.20082683. 19564350 PMC2715097

[25] Kim E, Kim M, Woo DH, Shin Y, Shin J, Chang N, Oh YT, Kim H, Rheey J, Nakano I, Lee C, Joo KM, Rich JN, Nam DH, Lee J 2013 Phosphorylation of EZH2 activates STAT3 signaling via STAT3 methylation and promotes tumorigenicity of glioblastoma stem-like cells. Cancer Cell 23:839–852. doi:10.1016/j.ccr.2013.04.008. 23684459 PMC4109796

[26] Ale A, Crepin VF, Collins JW, Constantinou N, Habibzay M, Babtie AC, Frankel G, Stumpf MP 2017 Model of host-pathogen interaction dynamics links *in vivo* optical imaging and immune responses. Infect Immun 85:e00606-16. doi:10.1128/IAI.00606-16. 27821583 PMC5203651

[27] Crepin VF, Habibzay M, Glegola-Madejska I, Guenot M, Collins JW, Frankel G 2015 Tir triggers expression of CXCL1 in enterocytes and neutrophil recruitment during *Citrobacter rodentium* infection. Infect. Infect Immun 83:3342–3354. doi:10.1128/IAI.00291-15. 26077760 PMC4534649

[28] Behnsen J, Jellbauer S, Wong CP, Edwards RA, George MD, Ouyang W, Raffatellu M 2014 The cytokine IL-22 promotes pathogen colonization by suppressing related commensal bacteria. Immunity 40:262–273. doi:10.1016/j.immuni.2014.01.003. 24508234 PMC3964146

[29] Ziesche E, Bachmann M, Kleinert H, Pfeilschifter J, Mu H 2007 The interleukin-22/STAT3 pathway potentiates expression of inducible nitric-oxide synthase in human colon carcinoma cells. J Biol Chem 282:16006–16015. doi:10.1074/jbc.M611040200. 17438334

[30] Rosenstiel P, Sina C, End C, Renner M, Lyer S, Till A, Hellmig S, Nikolaus S, Folsch UR, Helmke B, Autschbach F, Schirmacher P, Kioschis P, Hafner M, Poustka A, Mollenhauer J, Schreiber S 2007 Regulation of DMBT1 via NOD2 and TLR4 in intestinal epithelial cells modulates bacterial recognition and invasion. J Immunol 178:8203–8211. doi:10.4049/jimmunol.178.12.8203. 17548659

[31] Chan JM, Bhinder G, Sham HP, Ryz N, Huang T, Bergstrom KS, Vallance BA 2013 CD4^+^T cells drive goblet cell depletion during *Citrobacter rodentium* infection. Infect Immun 81:4649–4658. doi:10.1128/IAI.00655-13. 24101690 PMC3837981

[32] Jean Beltran PM, Federspiel JD, Sheng X, Cristea IM 2017 Proteomics and integrative omic approaches for understanding host-pathogen interactions and infectious diseases. Mol Syst Biol 13:92. doi:10.15252/msb.20167062. PMC5371729 28348067

[33] Liu Y, Zhang Q, Hu M, Yu K, Fu J, Zhou F, Liu X 2015 Proteomic analyses of intracellular *Salmonella enterica* serovar Typhimurium reveal extensive bacterial adaptations to infected host epithelial cells. Infect. Infect Immun 83:2897–2906. doi:10.1128/IAI.02882-14. 25939512 PMC4468536

[34] Pieper R, Fisher CR, Suh MJ, Huang ST, Parmar P, Payne SM 2013 Analysis of the proteome of intracellular *Shigella flexner*i reveals pathways important for intracellular growth. Infect Immun 81:4635–4648. doi:10.1128/IAI.00975-13. 24101689 PMC3837999

[35] Yang Y, Hu M, Yu K, Zeng X, Liu X 2015 Mass spectrometry-based proteomic approaches to study pathogenic bacteria-host interactions. Protein Cell 6:265–274. doi:10.1007/s13238-015-0136-6. 25722051 PMC4383758

[36] Pallett MA, Crepin VF, Serafini N, Habibzay M, Kotik O, Sanchez-Garrido J, Di Santo JP, Shenoy AR, Berger CN, Frankel G 2017 Bacterial virulence factor inhibits caspase-4/11 activation in intestinal epithelial cells. Mucosal Immunol 10:602–612. doi:10.1038/mi.2016.77. 27624779 PMC5159625

[37] Hardwidge PR, Rodriguez-Escudero I, Goode D, Donohoe S, Eng J, Goodlett DR, Aebersold R, Finlay BB 2004 Proteomic analysis of the intestinal epithelial cell response to enteropathogenic *Escherichia coli*. J Biol Chem 279:20127–20136. doi:10.1074/jbc.M401228200. 14988394

[38] Diamond DL, Syder AJ, Jacobs JM, Sorensen CM, Walters KA, Proll SC, McDermott JE, Gritsenko MA, Zhang Q, Zhao R, Metz TO, Camp DG, Waters KM, Smith RD, Rice CM, Katze MG 2010 Temporal proteome and lipidome profiles reveal hepatitis C virus-associated reprogramming of hepatocellular metabolism and bioenergetics. PLoS Pathog 6:e1000719. doi:10.1371/journal.ppat.1000719. 20062526 PMC2796172

[39] Pollard DJ, Berger CN, So EC, Yu L, Hadavizadeh K, Jennings P, Tate EW, Choudhary JS, Frankel G 2018 Broad-spectrum regulation of nonreceptor tyrosine kinases by the bacterial ADP-ribosyltransferase EspJ. mBio 9:e00170-18. doi:10.1128/mBio.00170-18. 29636436 PMC5893879

[40] Roumeliotis TI, Williams SP, Gonçalves E, Alsinet C, Del Castillo Velasco-Herrera M, Aben N, Ghavidel FZ, Michaut M, Schubert M, Price S, Wright JC, Yu L, Yang M, Dienstmann R, Guinney J, Beltrao P, Brazma A, Pardo M, Stegle O, Adams DJ, Wessels L, Saez-Rodriguez J, McDermott U, Choudhary JS 2017 Genomic determinants of protein abundance variation in colorectal cancer cells. Cell Rep 20:2201–2214. doi:10.1016/j.celrep.2017.08.010. 28854368 PMC5583477

[41] Medico E, Russo M, Picco G, Cancelliere C, Valtorta E, Corti G, Buscarino M, Isella C, Lamba S, Martinoglio B, Veronese S, Siena S, Sartore-Bianchi A, Beccuti M, Mottolese M, Linnebacher M, Cordero F, Di Nicolantonio F, Bardelli A 2015 The molecular landscape of colorectal cancer cell lines unveils clinically actionable kinase targets. Nat Commun 6:7002. doi:10.1038/ncomms8002. 25926053

[42] Sadanandam A, Lyssiotis CA, Homicsko K, Collisson EA, Gibb WJ, Wullschleger S, Gonzalez Ostos LC, Lannon WA, Grotzinger C, Rio D, Lhermitte B, Olshen AB, Wiedenmann B, Cantley LC, Gray JW, Hanahan D 2013 A colorectal cancer classification system that associates cellular phenotype and responses to therapy. Nat Med 19:619–625. doi:10.1038/nm.3175. 23584089 PMC3774607

[43] Azzam KM, Fessler MB 2012 Crosstalk between reverse cholesterol transport and innate immunity. Trends Endocrinol Metab 23:169–177. doi:10.1016/j.tem.2012.02.001. 22406271 PMC3338129

[44] Tall AR, Yvan-Charvet L 2015 Cholesterol, inflammation and innate immunity. Nat Rev Immunol 15:104–116. doi:10.1038/nri3793. 25614320 PMC4669071

[45] García JL, Uhía I, Galán B 2012 Catabolism and biotechnological applications of cholesterol degrading bacteria. Microbial Biotechnol 5:679–699. doi:10.1111/j.1751-7915.2012.00331.x. PMC3815891 22309478

[46] Caspi R, Billington R, Ferrer L, Foerster H, Fulcher CA, Keseler IM, Kothari A, Krummenacker M, Latendresse M, Mueller LA, Ong Q, Paley S, Subhraveti P, Weaver DS, Karp PD 2016 The MetaCyc database of metabolic pathways and enzymes and the BioCyc collection of pathway/genome databases. Nucleic Acids Res 44:D471–D480. doi:10.1093/nar/gkv1164. 26527732 PMC4702838

[47] Disson O, Blériot C, Jacob J-M, Serafini N, Dulauroy S, Jouvion G, Fevre C, Gessain G, Thouvenot P, Eberl G, Di Santo JP, Peduto L, Lecuit M 2018 Peyer’s patch myeloid cells infection by Listeria signals through gp38^+^ stromal cells and locks intestinal villus invasion. J Exp Med 215:2936–2954. doi:10.1084/jem.20181210. 30355616 PMC6219733

[48] Crepin VF, Collins JW, Habibzay M, Frankel G 2016 *Citrobacter rodentium* mouse model of bacterial infection. Nat Protoc 11:1851–1876. doi:10.1038/nprot.2016.100. 27606775

[49] Kilkenny C, Browne WJ, Cuthill IC, Emerson M, Altman DG 2010 Improving bioscience research reporting: the ARRIVE guidelines for reporting animal research. PLoS Biol 8:e1000412. doi:10.1371/journal.pbio.1000412. 20613859 PMC2893951

[50] Girard F, Batisson I, Frankel GM, Harel J, Fairbrother JM 2005 Interaction of enteropathogenic and Shiga toxin-producing *Escherichia coli* and porcine intestinal mucosa: role of intimin and Tir in adherence. Infect Immun 73:6005–6016. doi:10.1128/IAI.73.9.6005-6016.2005. 16113321 PMC1231093

[51] Tyanova S, Temu T, Sinitcyn P, Carlson A, Hein MY, Geiger T, Mann M, Cox J 2016 The Perseus computational platform for comprehensive analysis of (prote)omics data. Nat Methods 13:731–740. doi:10.1038/nmeth.3901. 27348712

[52] Cox J, Mann M 2012 1D and 2D annotation enrichment: a statistical method integrating quantitative proteomics with complementary high-throughput data. BMC Bioinformatics 13(Suppl 16):S12. doi:10.1186/1471-2105-13-S16-S12. PMC3489530 23176165

[53] Gregory Caporaso J, Kuczynski J, Stombaugh J, Bittinger K, Bushman FD, Costello EK, Fierer N, Peña AG, Goodrich JK, Gordon JI, Huttley G, Kelley ST, Knights D, Koenig JE, Ley RE, Lozupone C, Mcdonald D, Muegge BD, Pirrung M, Reeder J, Sevinsky JR, Turnbaugh PJ, Walters W, Widmann J, Yatsunenko T, Zaneveld J, Knight R 2010 QIIME allows analysis of high-throughput community sequencing data Intensity normalization improves color calling in SOLiD sequencing. Nat Methods 7:335–336. doi:10.1038/nmeth.f.303. 20383131 PMC3156573

[54] Callahan BJ, McMurdie PJ, Rosen MJ, Han AW, Johnson AJA, Holmes SP 2016 DADA2: High-resolution sample inference from Illumina amplicon data. Nat Methods 13:581–583. doi:10.1038/nmeth.3869. 27214047 PMC4927377

[55] Vizcaíno JA, Csordas A, del-Toro N, Dianes JA, Griss J, Lavidas I, Mayer G, Perez-Riverol Y, Reisinger F, Ternent T, Xu QW, Wang R, Hermjakob H 2016 2016 update of the PRIDE database and related tools. Nucleic Acids Res 44:D447–D456. 26527722 10.1093/nar/gkv1145 PMC4702828

