# Supplementary figures and images for: Intestinal Epithelial Cells and the Microbiome Undergo Swift Reprogramming at the Inception of Colonic Citrobacter rodentium Infection
Source: mBio. 2019 Apr 2;10(2):e00062-19. doi: 10.1128/mBio.00062-19 (PMC6445932; doi:10.1128/mBio.00062-19)

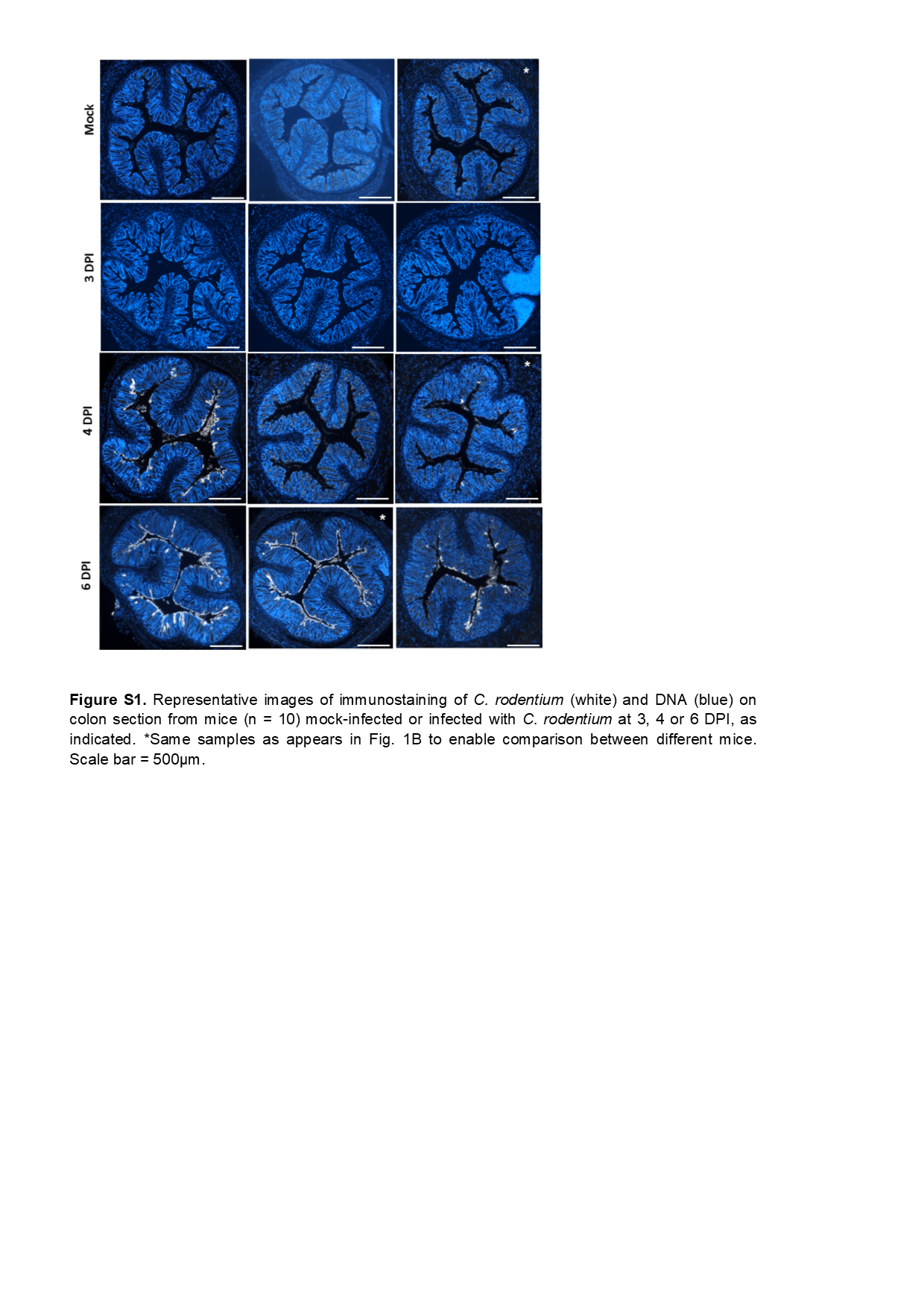

Supplement: FIG S1 [file mBio.00062-19-sf001.tif]

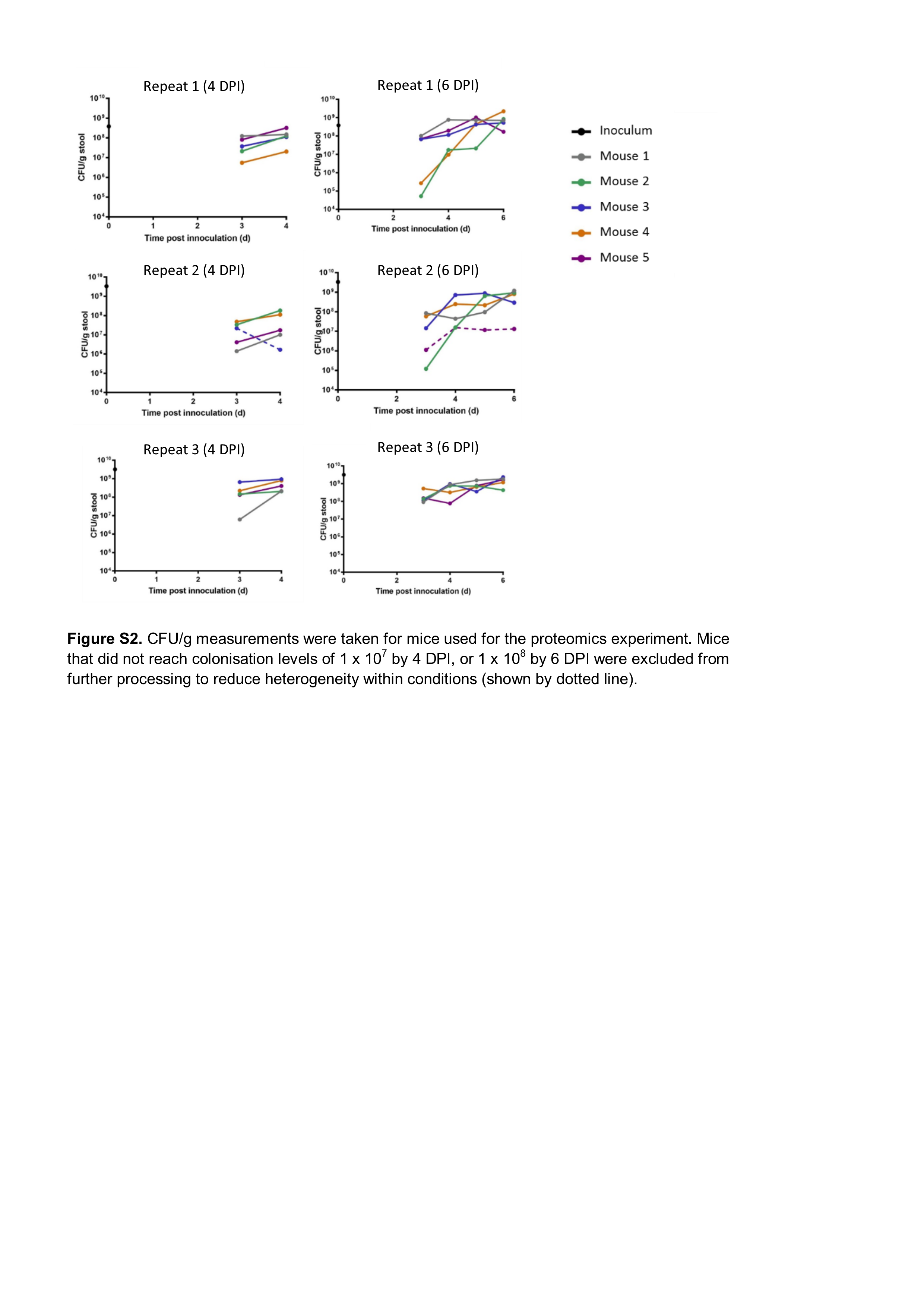

Supplement: FIG S2 [file mBio.00062-19-sf002.tif]

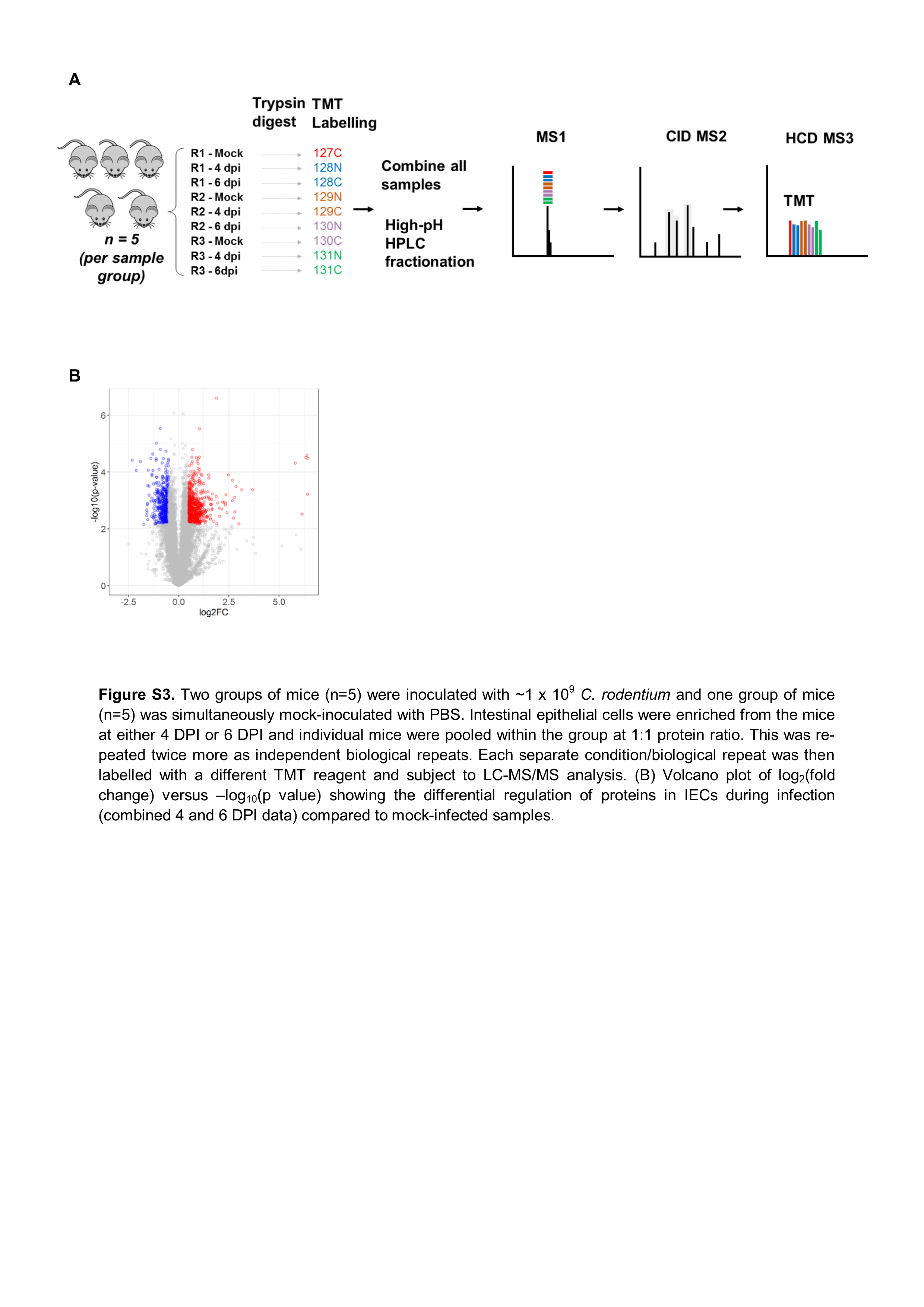

Supplement: FIG S3 [file mBio.00062-19-sf003.tif]

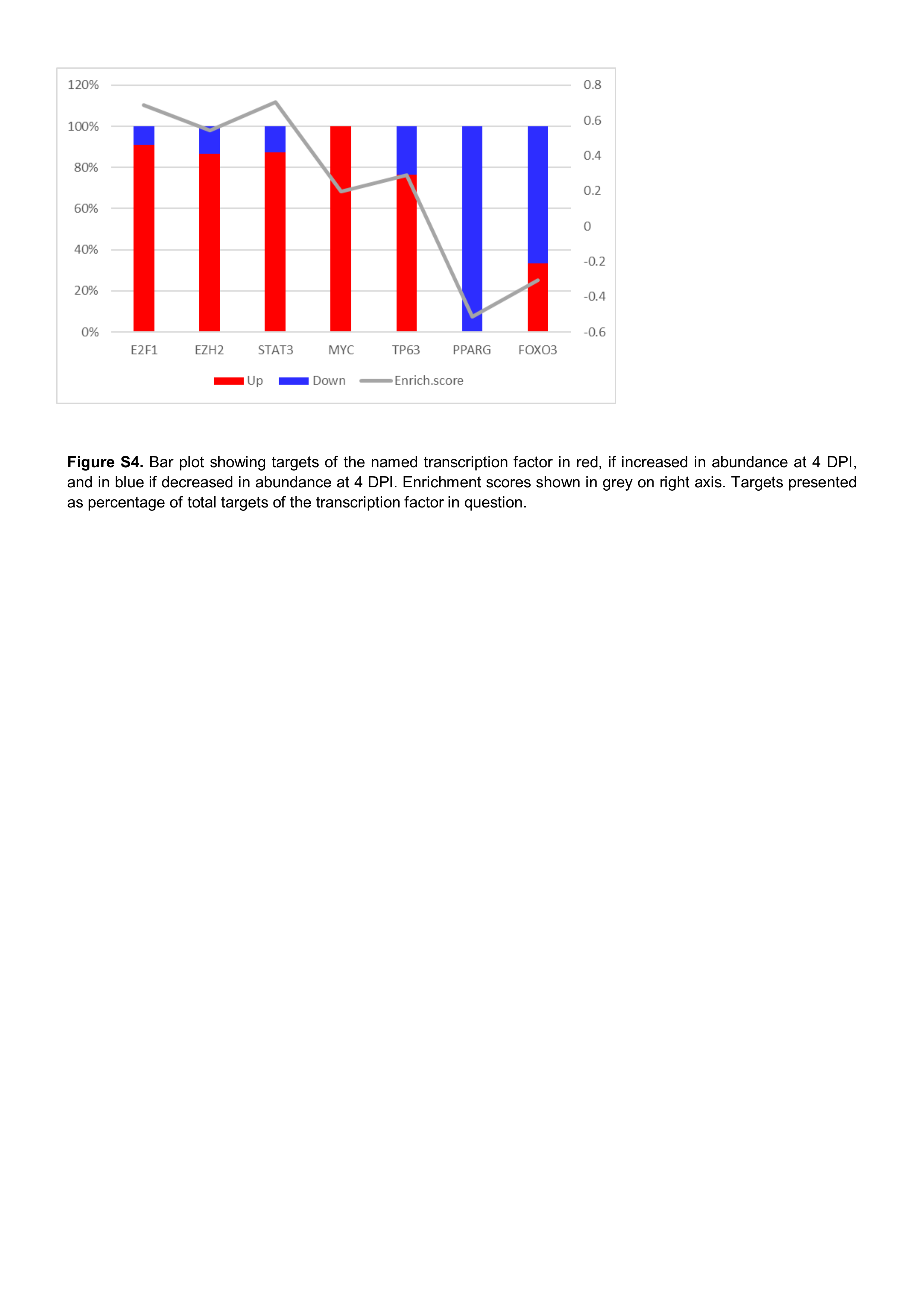

Supplement: FIG S4 [file mBio.00062-19-sf004.tif]

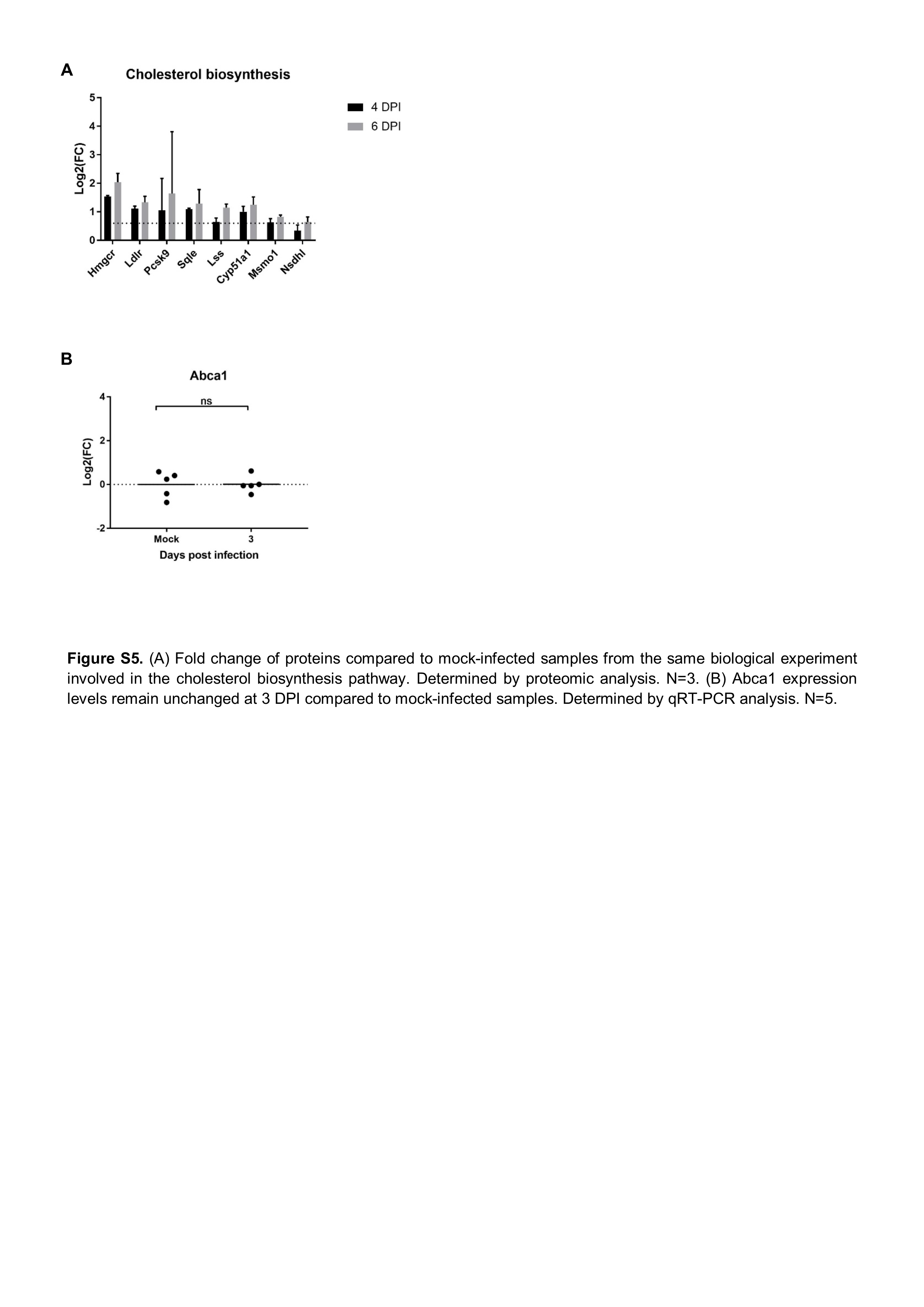

Supplement: FIG S5 [file mBio.00062-19-sf005.tif]

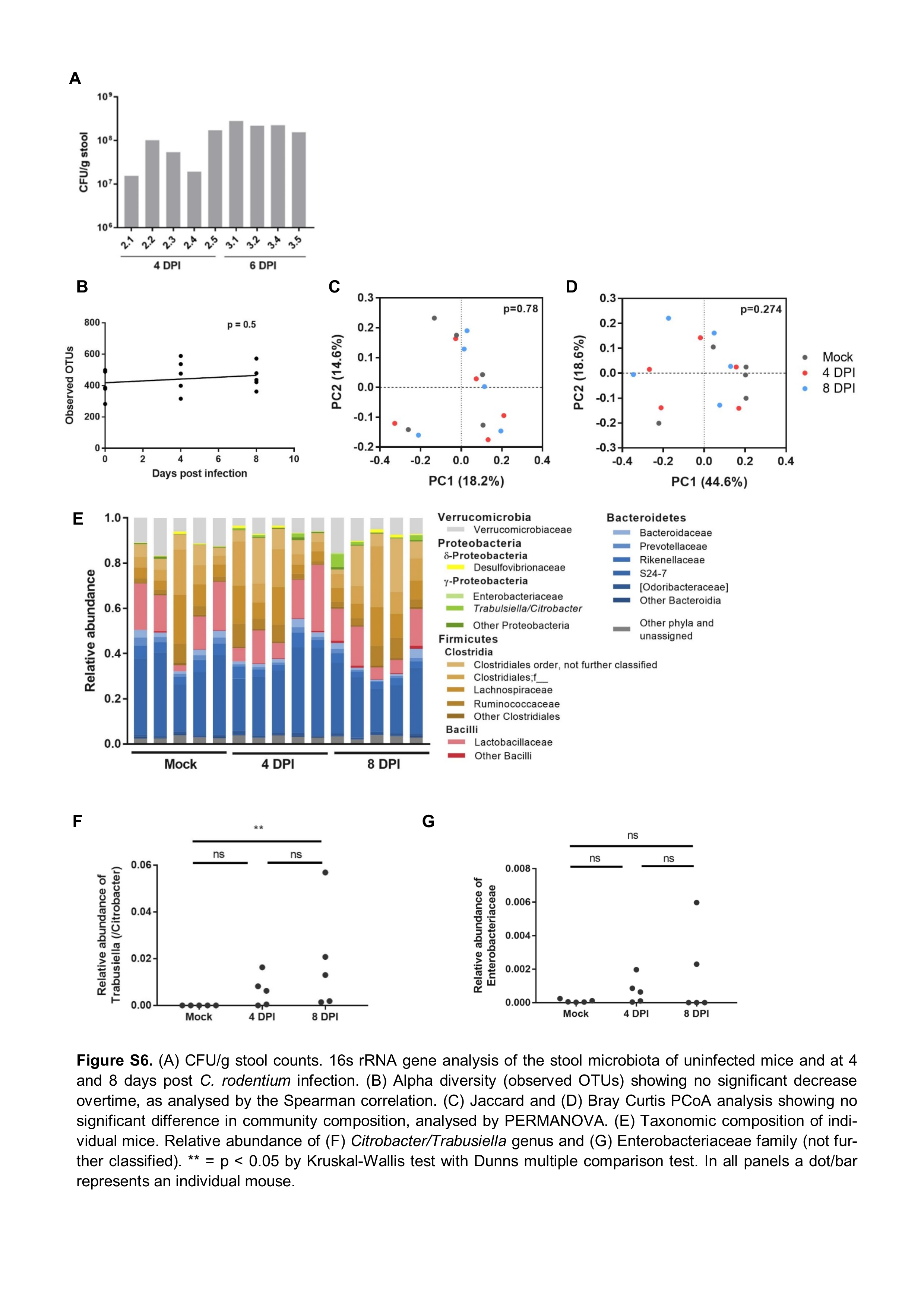

Supplement: FIG S6 [file mBio.00062-19-sf006.tif]

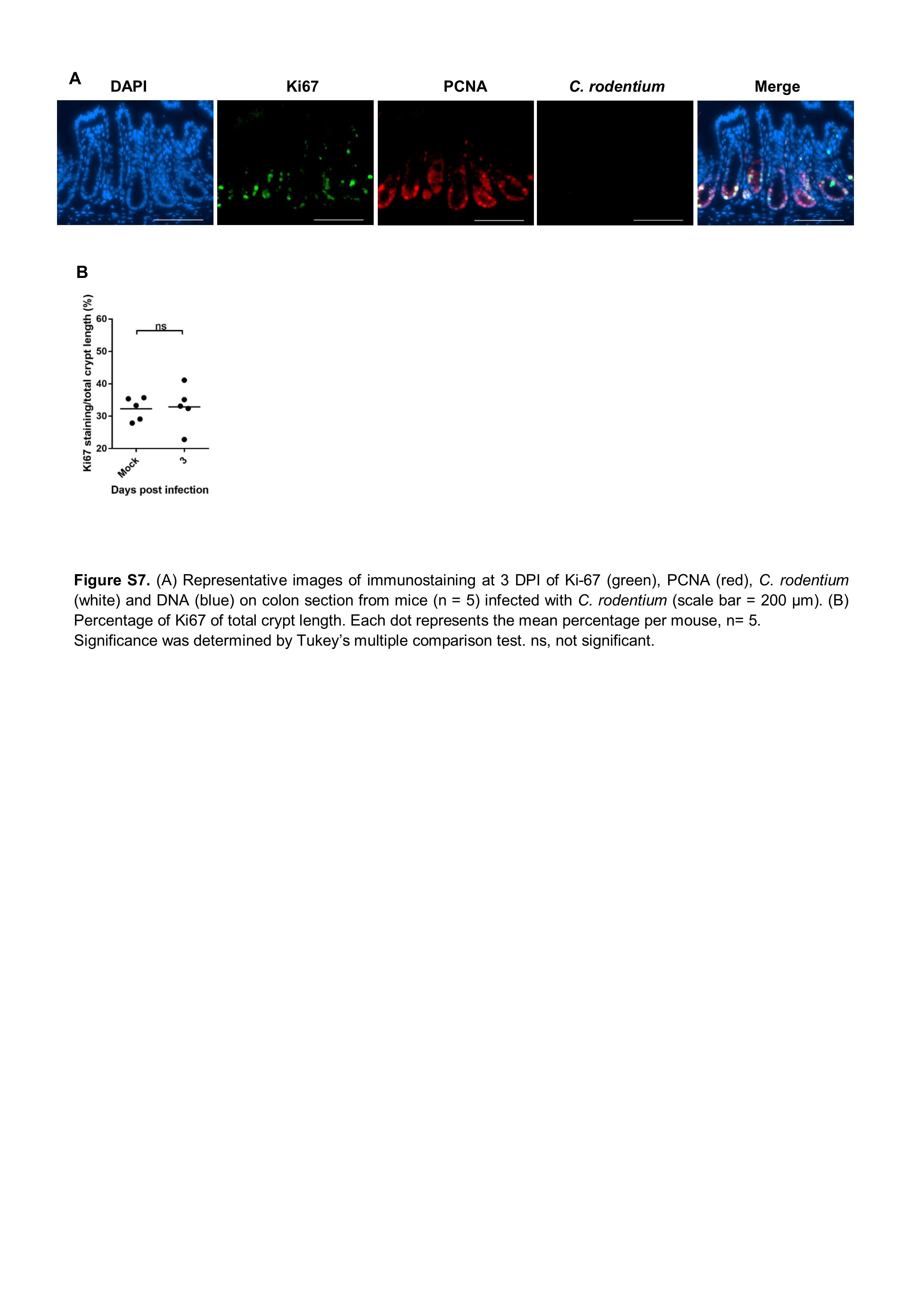

Supplement: FIG S7 [file mBio.00062-19-sf007.tif]

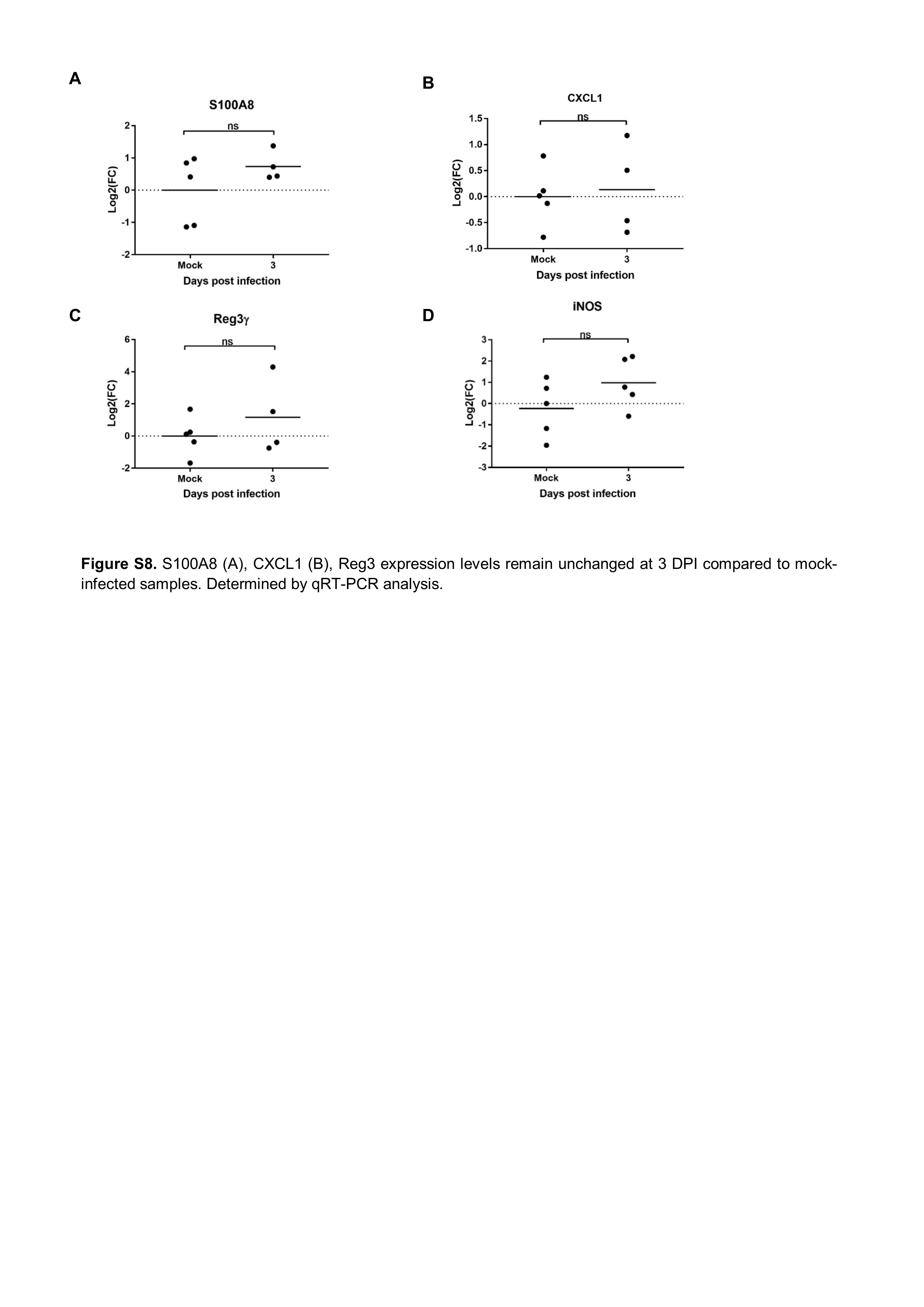

Supplement: FIG S8 [file mBio.00062-19-sf008.tif]
