# Supplementary material for: Intestinal Epithelial Cells and the Microbiome Undergo Swift Reprogramming at the Inception of Colonic Citrobacter rodentium Infection
Source: mBio. 2019 Apr 2;10(2):e00062-19. doi: 10.1128/mBio.00062-19 (PMC6445932; doi:10.1128/mBio.00062-19)
Supplement: TABLE S1 [file mBio.00062-19-st001.pdf]

Table S1. TMT-labels, pooled samples and mice selected for proteomics analysis. Mice that did not reach the colonisation threshold set were excluded from processing.

**Sample**	**1**	**2**	**3**	**4**	**5**	**6**	**7**	**8**	**9**
TMT label	127C	128N	128C	129N	129C	130N	130C	131N	131C
Group	R1-Mock	R1-4DPI	R1-6DPI	R2-Mock	R2-4DPI	R2-6DPI	R3-Mock	R3-4DPI	R3-6DPI
Individual mice	1.1<br>1.2<br>1.3<br>1.4<br>1.5	2.1<br>2.2<br>2.3<br>2.4<br>2.5	3.1<br>3.2<br>3.3<br>3.4<br>3.5	1.1<br>1.2<br>1.3<br>1.4<br>1.5	2.1<br>2.2<br>2.4<br>2.5	3.1<br>3.2<br>3.3<br>3.4	1.1<br>1.2<br>1.3<br>1.4<br>1.5	2.1<br>2.2<br>2.3<br>2.4<br>2.5	3.1<br>3.2<br>3.3<br>3.4<br>3.5
