# Supplementary material for: Intestinal Epithelial Cells and the Microbiome Undergo Swift Reprogramming at the Inception of Colonic Citrobacter rodentium Infection
Source: mBio. 2019 Apr 2;10(2):e00062-19. doi: 10.1128/mBio.00062-19 (PMC6445932; doi:10.1128/mBio.00062-19)
Supplement: TABLE S2 [file mBio.00062-19-st002.pdf]

Table S2. Primer sequences.

Primer Name	Primer Sequence
Ido1F_qPCR	CAATCAAAGCAATCCCCACTG
Ido1R_qPCR	AAAACGTGTCTGGGTCCAC
Cxcl1F_qPCR	AACCGAAGTCATAGCCACAC
Cxcl1R_qPCR	CAGACGGTGCCATCAGAG
Reg3gF_qPCR	TTACATCAACTGGGAGACGAATC
Reg3gR_qPCR	GGCCTTGAATTTGCAGACATAG
HmgcrF_qPCR	GCCCTCAGTTCAAATTCACAG
HmgcrR_qPCR	TTCCACAAGAGCGTCAAGAG
Slc5a9F_qPCR	ACTACATCCAATCTATCACCAGC
Slc5a9R_qPCR	ATCATACGCAGTATTCCGACG
Abca1F_qPCR	GGACATGCACAAGGTCCTGA
Abca1R_qPCR	CAGAAAATCCTGGAGCTTCAAA
GapdhF_qPCR	TCAACAGCAACTCCCACTCTTCCA
GapdhR_qPCR	ACCCTGTTGCTGTAGCCGTATTCA
S100a8F_qPCR	AGTGTCCTCAGTTTGTGCAG
S100a8R_qPCR	ACTCCTTGTGGCTGTCTTTG
Pcks9F_qPCR	TTTTATGACCTCTTCCCTGGC
Pcks9R_qPCR	ATTCGCTCCAGGTTCCATG
IdoLF_qPCR	ATGCTGTGCTATGTGACGAG
IdoLR_qPCR	TTTGCTCCCCGTGAACTG
Abcg5F_qPCR	CCTGAACATTCCAATCCCTTTG
Abcg5R_qPCR	ACGTTTCTATTTCCCGCTCTC
Reg4F_qPCR	CGCTGAGATGAACCCCAAG
Reg4R_qPCR	TGAGAGGGAAGTGGGAAGAG
Slc26a3F_qPCR	AGACGCACAGACATCACAAG
Slc26a3R_qPCR	TGTCACTGAGAAGCCATTCC
iNOSF_qPCR	CAG CTG GGC TGT ACA AAC CTT
iNOSR_qPCR	CAT TGG AAG TGA AGC GGT TCG
LdlrF_qPCR	ACCCGCCAAGATCAAGAAAG
LdlrR_qPCR	GCTGGAGATAGAGTGGAGTTTG
Dmbt1_qPCRF	GGAGGCTATGAGGACTATCTTTG
Dmbt1_qPCR	TGGTTTGGTCAGTTGGGTAG
